# Circulating proteomic signature of early death in heart failure patients with reduced ejection fraction

**DOI:** 10.1038/s41598-019-55727-1

**Published:** 2019-12-16

**Authors:** Marie Cuvelliez, Vincent Vandewalle, Maxime Brunin, Olivia Beseme, Audrey Hulot, Pascal de Groote, Philippe Amouyel, Christophe Bauters, Guillemette Marot, Florence Pinet

**Affiliations:** 1 Univ. Lille, CHU Lille, Inserm, Institut Pasteur de Lille, U1167 - RID-AGE - Facteurs de risque et déterminants moléculaires des maladies liées au vieillissement, F-59000 Lille, France; 2 FHU REMOD-HF, Lille, France; 3 Univ. Lille, CHU Lille, Inria Lille Nord-Europe, EA2694 - MODAL - MOdels for Data Analysis and Learning, F-59000 Lille, France; 4 Univ. Lille, « Institut Français de Bioinformatique », « Billille- plateforme de bioinformatique et bioanalyse de Lille », F-59000 Lille, France

**Keywords:** Gene regulatory networks, Prognostic markers

## Abstract

Heart failure (HF) remains a main cause of mortality worldwide. Risk stratification of patients with systolic chronic HF is critical to identify those who may benefit from advanced HF therapies. The aim of this study is to identify plasmatic proteins that could predict the early death (within 3 years) of HF patients with reduced ejection fraction hospitalized in CHRU de Lille. The subproteome targeted by an aptamer-based technology, the Slow Off-rate Modified Aptamer (SOMA) scan assay of 1310 proteins, was profiled in blood samples from 168 HF patients, and 203 proteins were significantly modulated between patients who died of cardiovascular death and patients who were alive after 3 years of HF evaluation (Wilcoxon test, FDR 5%). A molecular network was built using these 203 proteins, and the resulting network contained 2281 molecules assigned to 34 clusters annotated to biological pathways by Gene Ontology. This network model highlighted extracellular matrix organization as the main mechanism involved in early death in HF patients. In parallel, an adaptive Least Absolute Shrinkage and Selection Operator (LASSO) was performed on these 203 proteins, and six proteins were selected as candidates to predict early death in HF patients: complement C3, cathepsin S and F107B were decreased and MAPK5, MMP1 and MMP7 increased in patients who died of cardiovascular causes compared with patients living 3 years after HF evaluation. This proteomic signature of 6 circulating plasma proteins allows the identification of systolic HF patients with a risk of early death.

## Introduction

Heart failure (HF) is an important cause of mortality worldwide^1^. HF has different origins: non-ischaemic, such as cardiomyopathies, or ischaemic, after myocardial infarction (MI). Detection and treatment of HF are still unsatisfactory. Risk stratification of systolic HF patients is an important issue that can lead high-risk patients to invasive strategies New York Heart Association (NYHA) class, left ventricular ejection fraction (LVEF), B-type natriuretic peptide (BNP) level and peak exercise oxygen consumption (peak VO_2_) have been associated with the early death of HF patients^2,3^. However, this stratification needs to be improved. Prediction of mortality in HF patients using “conventional” prognostic evaluation showed moderate success and emphasized the requirement of models using a systems biology approach^4^. We previously performed proteomic profiling in a case/control study that had included patients with systolic HF. Forty-two differentially intense peaks were identified and used to develop proteomic scores. These scores allowed a better discrimination of HF patients^5^. Recently, a profiling by matrix-assisted laser desorption-ionization MS (MALDI-MS) showed that 14 peptides identified in plasma can predict clinical outcomes in HF patients^6^.

Recent advances in systems biology have opened new opportunities in the study of biomarker discovery and of the mechanistic context of HF. While previous analyses relied on the study of individual molecules, molecular network models allow the understanding of the complex mechanisms underlying HF. Systems-based approaches allow the identification of single biomarkers with mechanistic relevance or the identification of several biomarkers that could be used together^7^. Recently, a multimarker strategy showed that a model containing endothelin-1 (ET-1), N-terminal (NT)-proBNP, high-sensitivity troponin I (hsTnI) and soluble suppression of tumourigenicity 2 (sST2) are the best predictor of cardiovascular events, in a cohort of 115 patients with chronic systolic HF^8^. Given the complex physiology of HF, beyond BNP, novel biomarkers discovered by multimarker testing panels may supplement traditional clinical practice to further improve HF care^9^.

Aptamer-based approaches, such as the Slow Off-rate Modified Aptamer (SOMA) scan assay, have become a good alternative for biomarker discovery^10^. Indeed, the SOMAscan assay allows the quantification of a large set of proteins (more than one thousand) in plasma samples. Recently, it has been shown that the SOMAscan assay had excellent reproducibility for a majority of the measured proteins, which remained stable over a long period^11^. Moreover, the technical variability of the SOMAscan assay was very low, and its performance was quite robust^12^. The SOMAscan assay has already been used to identify new biomarkers in several pathologies, such as cancers^13^, Alzheimer’s disease^14^, influenza^15^, coronary heart disease^16^ and cardiovascular diseases^15^. Recently, a proteomic signature of age, identifying 76 proteins correlated with chronological age, was characterized using the SOMAscan assay^17^.

The aim of the present study was to identify circulating plasma proteins that could predict the early death of HF patients, within 3 years of follow-up after hospitalization for systolic HF. The subproteome targeted by the SOMAscan assay was profiled in plasma samples from 168 HF patients with reduced LVEF selected from the “INsuffisant CArdiaque” (INCA) study^2^. A molecular network model was built based on significantly modulated proteins in the plasma of HF patients to study the molecular mechanisms underlying HF. A statistical penalized regression analysis was performed to take into account the high number of variables compared to the available number of individuals, and it identified six proteins that could predict the early death of HF patients. Finally, 4 of the 6 identified proteins were measured and validated by conventional assays in the plasma of a subset of 66 HF patients with a 3-year follow-up.

## Results

### INCA population characteristics

Table 1 shows the baseline characteristics of the patients included in the INCA population^2,5^. Patients in the two groups were matched for age, sex and HF aetiology. Patients who died of cardiovascular (CV) death after 3 years had higher NYHA class, higher BNP level, higher creatinine level and lower peak VO_2_ compared to patients who lived. Three years follow-up seems appropriate as it is relatively close to the HF evaluation but sufficient for an expected number of cardiovascular deaths that could be analysed as previously described^18^.

**Table 1:** Baseline characteristics of the patients included in the INCA study.

	Cardiovascular death (Case, (n = 84))	No cardiovascular death (Control, n = 84))	*P* value^≠^
Age (years)	58.73 ± 10.67	59 ± 10	1
Male	78	78	1
**HF aetiology**			
Ischaemic Non-ischaemic	51 33	51 33	1
Diabetes mellitus	53	51	0.874
**NYHA class**			
1 2 3	1 52 31	8 61 15	0.003
LV ejection fraction (%)	28.11 ± 9.95	29.30 ± 9.20	0.440
Peak VO_2_ (mL/min/kg)	10.5 ± 1.73	21 ± 5.19	1.933E-07
BNP*			
Low Intermediate High	14 38 29	36 25 20	4.550E-04
Creatinine (mg/L)	12.55 ± 3.55	11.12 ± 2.63	0.003
**Treatment at inclusion**			
ACE inhibitors β-Blockers Diuretics	77 76 75	77 78 65	1 0.577 0.038

^*^BNP was measured by a radio-immuno-assay (Shionoria BNP kit, Shionogi & Co. Ltd., Osaka, Japan) from 1998 to 2003 and by the Triage BNP assay (Biosite diagnostics Inc., San Diego, CA, USA) from 2003 to 2005. The BNP level was categorized as low (deciles 1, 2 and 3), intermediate (deciles 4, 5, 6 and 7) or high (deciles 8, 9 and 10) for each individual patient. Continuous variables are expressed as mean ± standard deviation (SD), and the means of the two groups were compared using Student’s t-test. Categorical variables are presented as absolute number and/or percentages whose distribution between the two groups was compared using the χ2 test or the Fisher test, as appropriate.

### SOMAscan assay identified modulated proteins in the plasma of HF patients who died of CV causes

**Figure 1:**
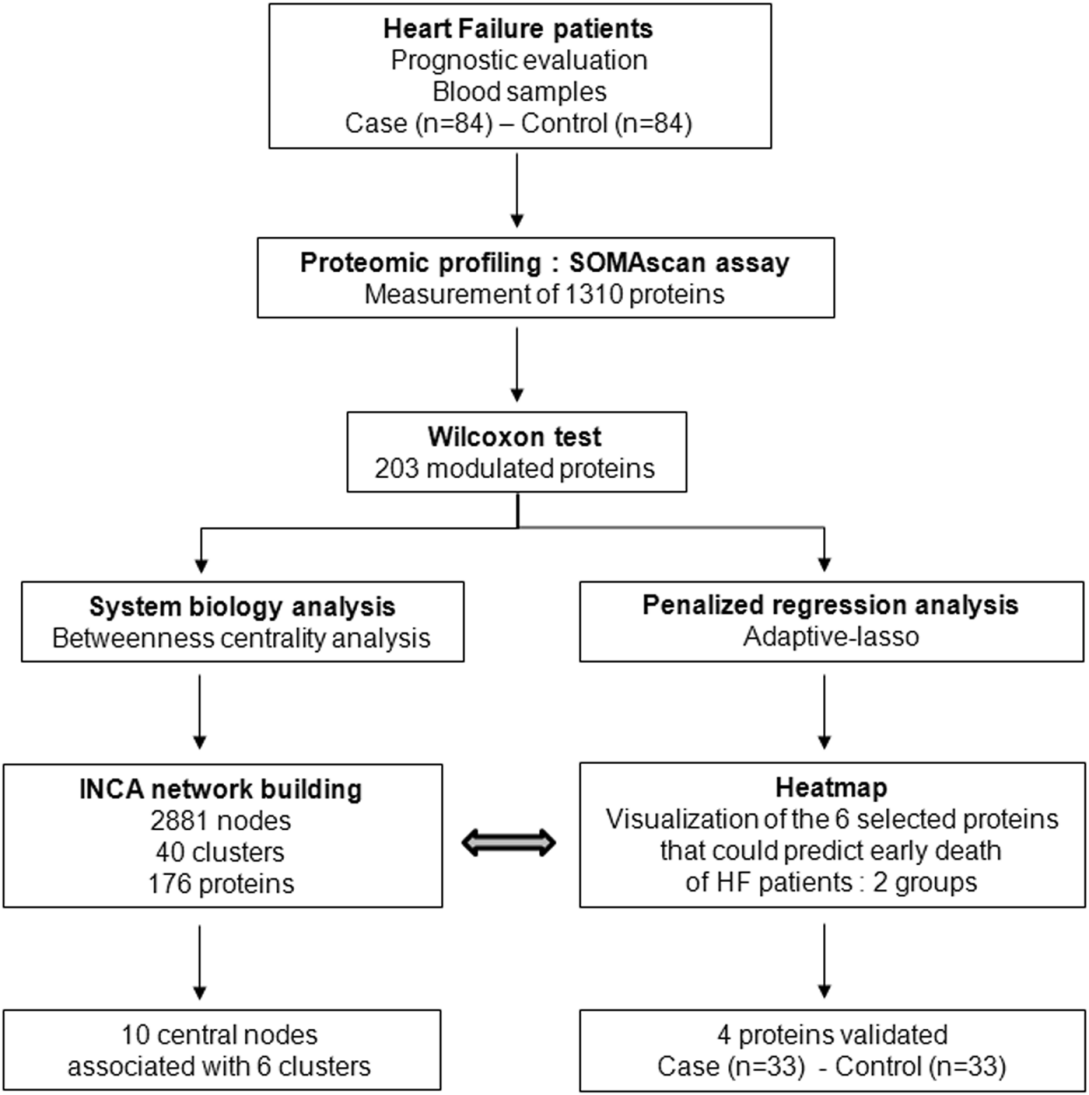
Overview of the study. Patients with systolic HF evaluation from the INCA study (84 with CV death (cases) and 84 alive (controls) were selected for measurement of 1310 plasma proteins by SOMAscan assay. By the Wilcoxon test, we identified 203 modulated proteins between patients who died of CV causes and the patients who were alive 3 years after HF evaluation. A molecular network was built based on these 203 proteins (see Supplementary Table S1). In parallel, we used an adaptive LASSO on the 203 modulated proteins. Six proteins were selected and linked to the INCA network built. Four proteins were measured by conventional assays (ELISA and Luminex technology) for validation in a subpopulation (33 cases and 33 controls).

In the HF patients of the INCA study, 1310 proteins were quantified using the SOMAscan proteomic profiling platform. In total, 203 proteins were significantly modulated in the plasma of patients who died during the 3-year follow-up compared to patients who were alive, using a cutoff of adjusted p-value < 0.05 and absolute log2 fold change (FC) above 0.25 (Supplementary Table S1). As shown in Fig. 1, we then used 2 strategies for analysing our high-throughput proteomic profiles. First, we built a molecular network to study the pathophysiological mechanisms underlying HF, and second, we assessed the performance of proteomic models to identify proteins that could predict the early death of HF patients after HF evaluation during hospitalization.

### The INCA molecular network model revealed the mechanistic context underlying HF

The INCA molecular network model was built based on the 203 proteins that were abundant (log2FC > 0.25) and significantly modulated in the plasma of INCA patients. The 203 significantly changed proteins could be mapped to 211 nodes, which were used as “seed nodes” to construct the network model. Proteins differentially expressed were embedded in a context of known molecular interactions (Supplementary Table S2) and analysed for mechanistic aspects using a molecular interaction network modelling approach. The resulting full INCA network contained 2881 nodes, including 1639 proteins, 1072 microRNAs and 170 metabolites linked by 15,061 edges (Supplementary Table S3) describing relationships between nodes.

**Figure 2:**
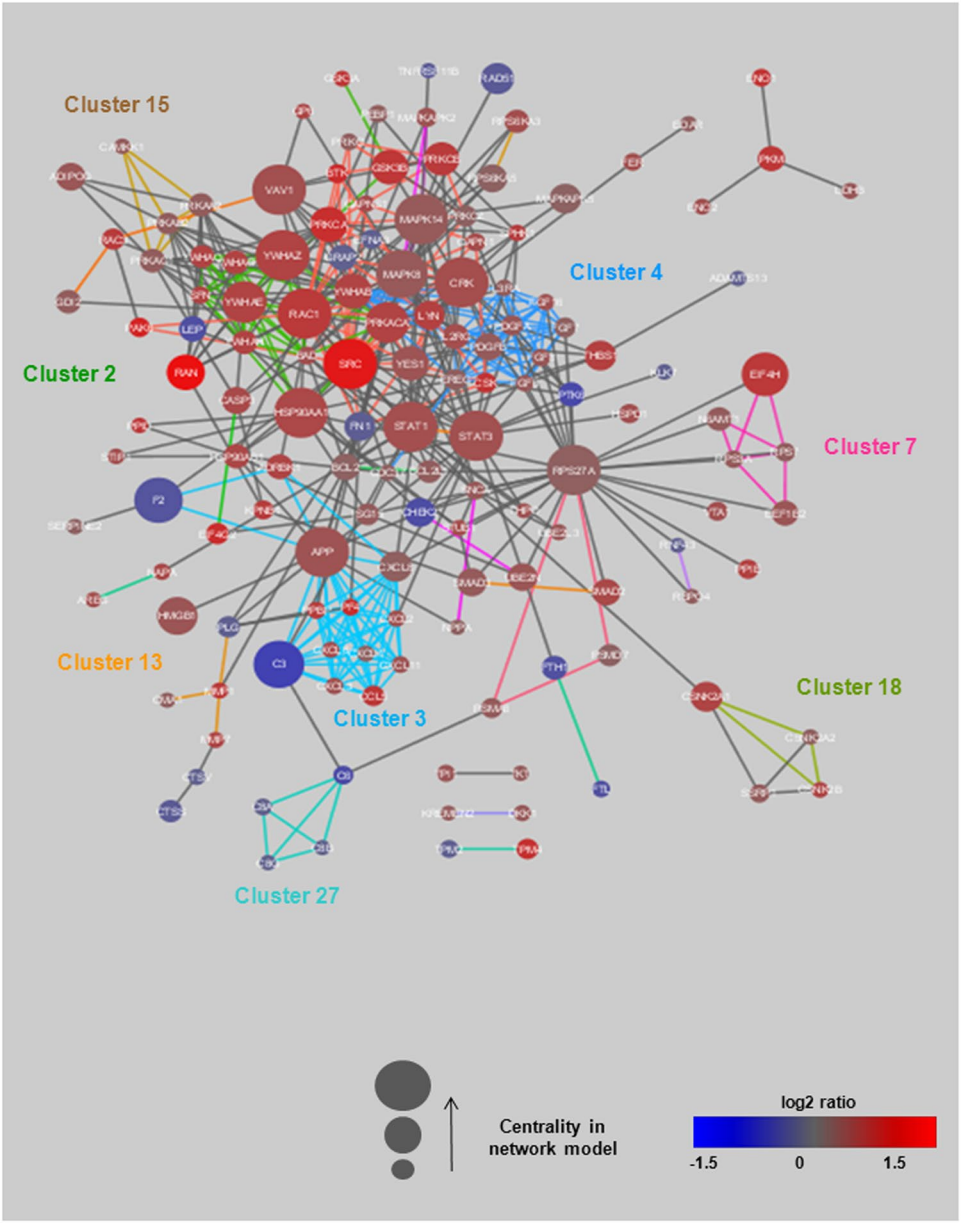
Subnetwork of the INCA molecular network model containing seed nodes that are directly interacting. This INCA subnetwork includes all significantly changed proteins (seed nodes) and their direct interactions for 8 clusters (1, 2, 3, 4, 7, 13, 15 and 27). Node size is scaled by the betweenness centrality in the full network model, meaning that a higher size corresponds to a higher centrality. The colour of the nodes represents the log2FC (or log2 ratio) of the comparison between the “case” and “control” groups. Proteins, microRNAs (miRNAs) and metabolites are indicated by different forms. Blue colour indicates decreased expression, and red colour indicates increased expression in patients who died of CV causes. Edges are coloured by cluster assignment in the network model (for corresponding pathway annotations, see Supplementary Table S4).

To assess the mechanistic context associated with the proteins, the INCA molecular network model was clustered based on its topology, and clusters were functionally annotated to biological pathways by Gene Ontology (http://geneontology.org/). Clusters are described as a group of nodes that are more highly connected to each other than to other nodes in the network. Forty clusters were identified in the INCA molecular network model. Thirty-four of these clusters could be annotated to at least one pathway (Supplementary Table S4). In total, 176 INCA proteins were part of one cluster. The most enriched clusters were associated with the immune system (clusters 1 and 6), transcription and translation (clusters 5 and 7) and signalling pathways (clusters 3, 4, 10 and 11). Figure 2 shows one of the INCA subnetworks, which included all significantly changed proteins (seed nodes) and their direct interactions for 8 clusters (1, 2, 3, 4, 7, 13, 15 and 27).

The number of shortest paths connecting any 2 nodes in the network defines the betweenness centrality of the node. The centrality of each node was calculated to assess the relevance of individual molecules in the INCA network model. A high centrality indicates a crucial role of the node in the INCA network model, while a low centrality suggests a more peripheral role. Nodes displaying a high centrality have been shown to be potential biomarkers^7^, suggesting that proteins with a high betweenness centrality in the INCA network may be potential biomarkers of HF. Interestingly, the 10 most central nodes, corresponding to molecules with the highest centrality in the INCA network, were mainly proteins measured in the INCA population by the SOMAscan assay and were associated with 6 clusters (Table 2): cluster 1 was related to immune response, cluster 2 to the plasma membrane, cluster 5 to DNA methylation, cluster 9 to the cell cycle, cluster 10 to G protein signalling and cluster 11 to the JAK-STAT cascade. Furthermore, some of these proteins, such as STAT1 and STAT3, are known to play a role in cardiovascular diseases^19^.

**Table 2:** Top 10 nodes with the highest betweenness centrality in the INCA network model.

Name/UniProt ID*	Full name of proteins	Betweenness Centrality^≠^	Cluster^≠^	Best pathways^‡^
RPS27A/P62979	Ribosomal protein S27a	0.097	9	Cell cycle
STAT3/P40763	Signal transducer and activator of transcription 3	0.071	11	JAK-STAT cascade
MK14/Q16539	Mitogen-activated protein kinase 14	0.062	5	DNA methylation
RAC1/P63000	Ras-related C3 botulinum toxin substrate 1	0.060	1	Immune response
HSP90A/P07900	Heat shock protein 90 kDa alpha family class A member 1	0.050	2	Plasma membrane
STAT1/P42224	Signal transducer and activator of transcription 1	0.049	11	JAK-STAT cascade
SRC/P12931	SRC proto-oncogene	0.047	1	Immune response
VAV1/P15498	Vav guanine nucleotide exchange factor 1	0.045	10	G protein signalling
1433Z/P63104	Tyrosine 3-monooxygenase/tryptophane 5-monooxygenase activation protein, zeta	0.044	2	Plasma membrane
CRK/P46108	V-crk avian sarcoma virus CT10 oncogene homologue	0.042	1	Immune response

*Name and UniProt ID are provided from the UniProtKB database (https://www.uniprot.org); ≠ Information provided from the INCA network and supplementary Table S4; ^‡^Information provided by GO (Gene Ontology, http://geneontology.org/).

### Selection of 6 candidate proteins that predict early death of HF patients and visualization of their modulation

The penalized regression analyses were performed on the 203 proteins that were significantly modulated between the two groups of patients. As explained in the Materials and Methods section, to limit the influence of potential extreme individuals, we considered all possible subsets of individuals in our 168-patient sample by removing one individual in each subset, thus resulting in 168 different subsets. For each subset of individuals, a particular subset of variables was selected using the adaptive Least Absolute Shrinkage and Selection Operator (LASSO). We kept only the proteins whose frequency of selection by the adaptive LASSO was higher than 0.90 among the 168 different training subsets. This analysis allowed the selection of 6 modulated proteins between the two groups, patients who died of CV causes and patients alive after 3 years: Complement C3 (C3), mitogen-activated protein kinase-activated protein kinase 5 (MAPK5), cathepsin S (CATS), matrix metallopeptidase 1 (MMP1), matrix metallopeptidase 7 (MMP7) and family with sequence similarity 107 member B (F107B) (Table 3).

**Table 3:** List of the 6 candidate proteins selected by LASSO analysis.

Protein full name (Protein) UniProt ID*	Fold-change (Case *vs*. Control, mean ± SEM)**	*P* value^≠^	Frequency^‡^	Betweenness centrality/Rank^≈^	Cluster in INCA network	Best GO pathways^≡^
Complement C3 (C3) P01024	0.65 (104221 ± 4830 *vs*.159620 ± 5910)	3. 18 10^−7^	1	0.037/13	3	Protein G
Mitogen-activated protein kinase-activated protein kinase 5 (MAPK5) Q8IW41	1.17 (484.3 ± 15.5 *vs*. 413.6 ± 10.6)	0.0066	0.97	0.017/30	6	Diseases of immune system
Cathepsin S (CATS) P25774	0.78 (752.6 ± 16.1 *vs*. 965.9 ± 25.2)	1.38 10^−6^	1	0.008/63	9	Cell cycle
Matrix metallopeptidase 1 (MMP1) P03956	1.96 (1499.6 ± 93.7 *vs*. 766.4 ± 40.2)	5.49 10^−5^	1	0.001/202	13	Extracellular matrix organization
Matrix metallopeptidase 7 (MMP7) P09237	1.44 (913.1 ± 51.6 *vs*. 635.1 ± 41.5)	0.0011	1	0.001/213	13	Extracellular matrix organization
Family with sequence similarity 107 member B (F107B) Q9H098	0.84 (812.4 ± 21 *vs*. 972.4 ± 28.2)	5.54 10^−4^	1	0.0004/353	—	—

^*^Full name, protein symbol and UniProt ID are provided from the UniProtKB database (https://www.uniprot.org/); **Data are expressed in relative fluorescence units; ^≠^*P* value was calculated by the Mann-Whitney-Wilcoxon test; ^‡^Frequency of selection after the 168 adaptative LASSOs; ^≈^Information provided from the INCA network (for more details, see Supplementary Fig. S1, and Table S4); ^≡^Information provided by GO (Gene Ontology, http://geneontology.org/); -, no cluster and information available on the INCA network.

**Figure 3:**
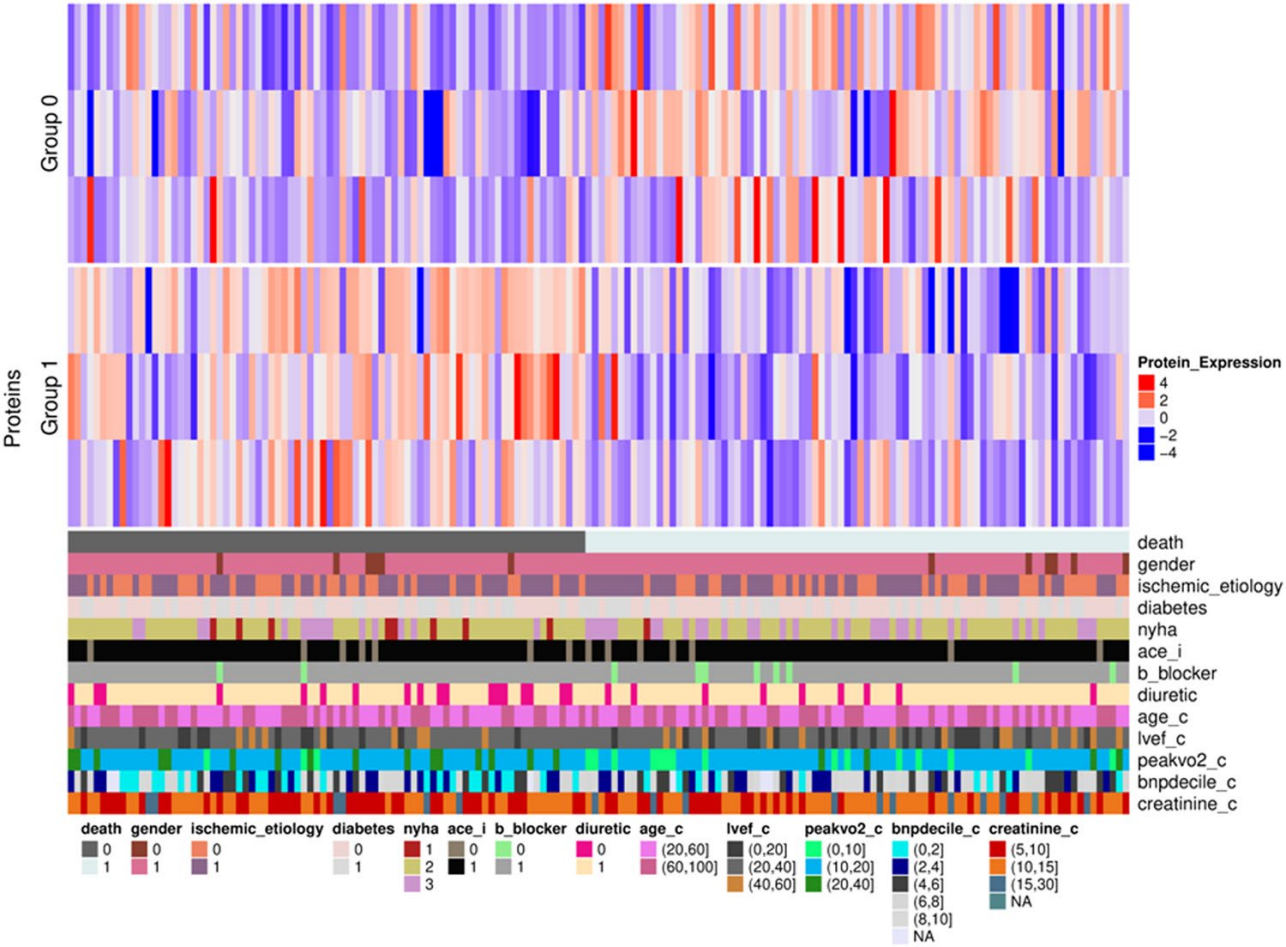
Heat map visualization of the 6 identified and selected proteins. Columns represent the patients divided into 2 fixed groups (group 0: control; and group 1: case). Rows above the patients represent the 6 proteins that were gathered based on their expression profile. Cells are coloured based on the protein abundance. Red represents a high abundance, while blue indicates a low abundance. The coloured bars below the patients represent the different clinical parameters detailed in Table 1 with 0 = no and 1 = yes, except for sex (0 = women and 1 = men) or when values are indicated. ace_i: angiotensin-converting enzyme inhibitor.

A heat map was drawn for these 6 selected proteins, which visualized sub-groups of proteins with similar expression profiles in each group of patients. As shown in Fig. 3, 2 sub-groups of proteins were identified. Both sub-groups contained 3 proteins: Group 0 contained 3 proteins increased in the plasma of patients who died of CV causes compared to patients who were alive after 3 years of follow-up (MAPK5, MMP1 and MMP7), while group 1 contained 3 proteins decreased in the plasma of patients who died of CV causes (C3, CATS and F107B). All proteins had a log2FC > 0.25 between the 2 groups of patients, a frequency > 0.9 and a significant ANOVA *P*-value (<0.05).

**Figure 4:**
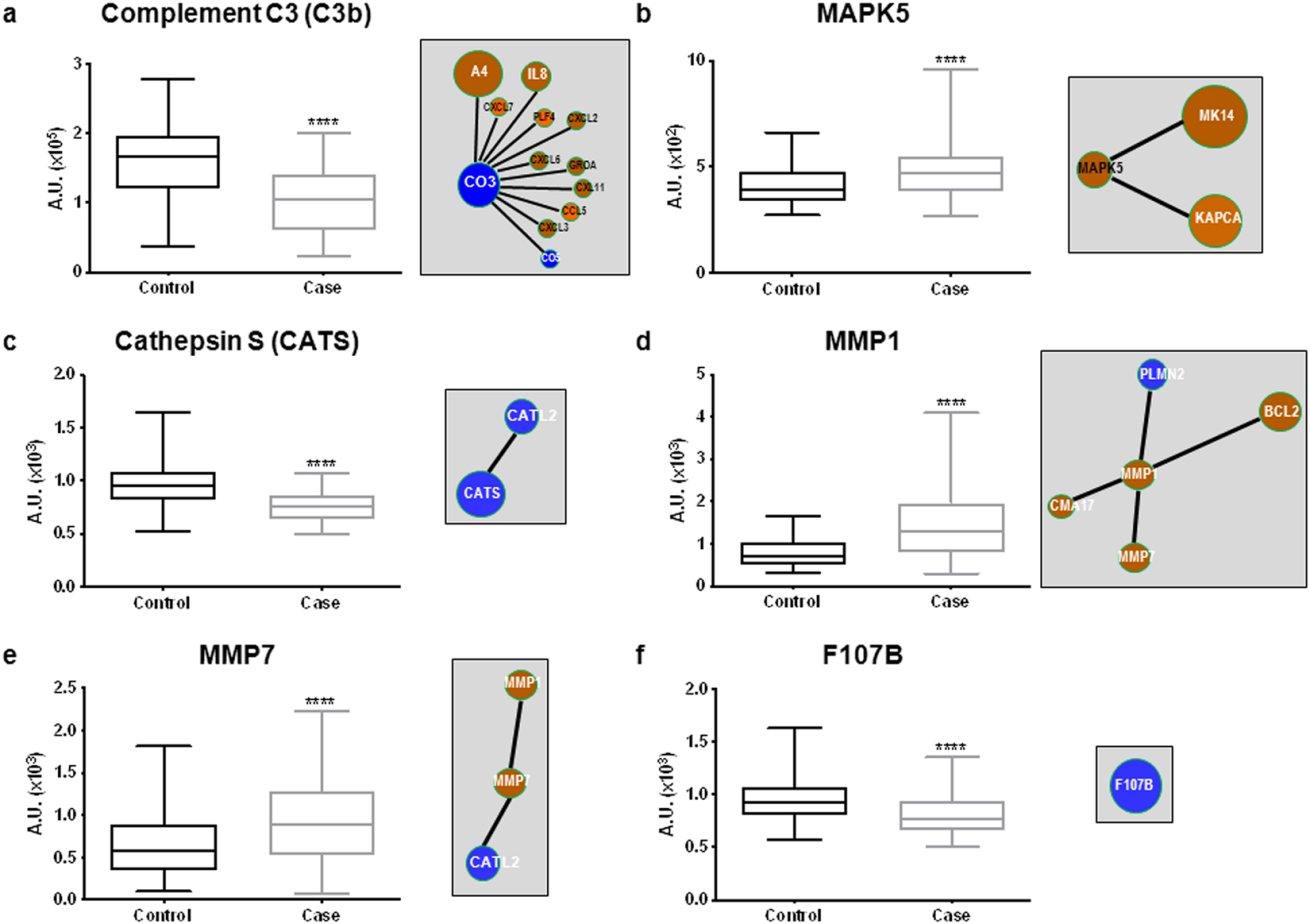
Plasma levels of the 6 proteins quantified by Somalogic and linked to the molecular INCA network. Quantification of complement C3b (**a**), MAPK5 (**b**), cathepsin S (**c**) MMP1 (**d**), MMP7 (**e**) and F107B (**f**) by SOMAscan assay (left panels) and closest edges from the INCA network (right panels). Data are expressed in arbitrary units (AU) corresponding to relative fluorescence units for the SOMAscan assay. Data are presented as box-and-whisker plots showing median (line) and min to max (whisker). Statistical significance was determined by the Wilcoxon-Mann-Whitney test. ****P < 0.0001. Visualization of the INCA molecular subnetworks centralized on these molecules (right panels) for their interactions with other proteins quantified in the plasma of INCA patients. The colour of the nodes represents the log2FC of the comparison between the 2 groups of patients who died of CV causes (case) or alive (control) after 3 years, with red corresponding to high log2FC and blue to low log2FC (for details, see Supplementary Table S5). The size of the nodes is related to the centrality calculated from the INCA network model.

By construction, the 6 proteins selected by the adaptive LASSO approach were all seed nodes in the INCA molecular network (Supplementary Table S4), and quantification by the SOMAscan assay for these 6 proteins is detailed (Fig. 4). Their centrality, corresponding rank and cluster in the INCA network are listed in Table 3. Three of them, C3, MAPK5 and CATS, had a high centrality, suggesting an important role of these proteins in the mechanisms underlying HF.

Targets associated with the 6 proteins in the INCA molecular network are indicated in the corresponding panels (Fig. 4), and detailed information about their abundance from the SOMAscan assay is provided (Supplementary Table S5). The 6 proteins were part of 4 different clusters of the INCA network: C3 belonged to cluster 3, associated with G protein signalling; MAPK5 belonged to cluster 6, associated with diseases of the immune system; CATS belonged to cluster 9, associated with the cell cycle; and MMP1 and MMP7 belonged to cluster 13, associated with extracellular matrix organization. F107B was not assigned to any cluster (Supplementary Table S5). Interestingly, all the targets linked in the INCA network to CATS and MAPK5 were regulated significantly in the same manner. Two targets, PLMN and CATL2, linked in the INCA network to MMP1 and MMP7, were oppositely regulated but both are in cluster 13 in which 51 nodes are associated to 91 edges related to regulation of extracellular matrix (activation/degradation). Except for C6, all the targets related to C3 (downregulated in patients who died of CV causes) in the cluster 3 of INCA network were upregulated (Supplementary Table S5). This cluster contains 140 nodes associated with 1020 edges associated with several functions with C3 and C6 associated with the same GO term (regulation of complement).

### Quantification of C3, CATS, MMP1 and MMP7 in a subset of the INCA population

To assess the relevance of the results obtained by the SOMAscan assay, we were only able to quantify C3, CATS, MMP1 and MMP7 in the plasma of patients from a subset of INCA patients by conventional assays: C3, MMP1 and MMP7 were measured using Luminex technology, while CATS was quantified by enzyme-linked immunosorbent assay (ELISA). We did not find any adequate and specific assays for MAPK5 and F107B. As shown in Supplementary Fig. S1, we obtained consistent data between the SOMAscan assay (left panels) and conventional assays (middle panels), except for CATS (Supplementary Fig. S1d). C3 was significantly decreased in the plasma of patients who died of CV causes compared to patients who were alive after 3 years of follow-up (*P* = 0.038) (Supplementary Fig. S1a). MMP1 levels were significantly increased in the plasma of patients who died of CV causes (*P* = 0.003) (Supplementary Fig. S1b). Finally, MMP7 plasma levels were very low, and it was not possible to calculate concentrations for all the samples, so we compared the fluorescence intensity value for all the samples in the 2 groups of patients. MMP7 was significantly increased in the plasma of patients who died of CV causes (*P* = 0.016) (Supplementary Fig. S1c). We did not validate the decrease in CATS in the plasma of patients who died of CV causes after 3 years of follow-up (*P* = 0.311) and observed a higher variability (min and max) compared to the SOMAscan assay (Supplementary Fig. S1d). However, 2 isoforms of CATS have been described (Supplementary Fig. S2), and we could speculate that one of the assay measured the 2 isoforms of CATS; this might explain the discrepancy between the 2 assays. The 4 proteins were also tested for the correlation between the SOMAscan data and the conventional assay data (Supplementary Fig. S2, right panels). Interestingly, only the levels of MMP1 and MMP7 were significantly correlated in both assays. As expected, CATS was not correlated, nor was C3. The latter might be explained by the fact that there are specific SOMAmers to detect C3a (SL000313) and C3b (SL00314), and the conventional assay did not discriminate between C3a and C3b.

### Correlation of the candidate proteins with BNP and peak VO_2_

Finally, we looked for correlations of the 6 candidate proteins with traditional markers in patients with CV diseases (Supplementary Fig. S3). No significant correlations were found for MAPK5, MMP7 or CATS. Two candidate proteins were significantly negatively correlated with BNP measured at the hospital, C3b (r = −0.242) and F107B (r = −0.198), and a trend was found for MMP1 (r = 0.1543, *P* = 0.059). Interestingly, a significant positive correlation was observed for C3b (r = 0.319), CATS (r = 0.227) and F107B (r = 0.206) with the peak VO_2_, and there was a significant negative correlation for MMP1 (r = −0.282) and MMP7 (r = −0.204) (Supplementary Fig. S3).

## Discussion

With population ageing, HF remains a public health issue. It is important to improve the risk stratification of HF patients to lead patients to the most suitable treatment. The aim of this study was to identify a set of plasma proteins that could predict the early death of HF patients (within 3 years of follow-up) using systems biology analysis and statistical approaches on high-throughput proteomic data.

For this purpose, we used the INCA prospective cohort, which included patients with systolic HF (mean age 58-59 years)^2,5^ to determine a proteomic signature with circulating biomarkers of early death (3 years after hospitalization for HF). We have chosen to use aptamer-based technologies as proteomic platforms because this technology allowed the highly specific quantification of many proteins, allowing the building of molecular networks to discover biomarkers of different diseases, including cardiovascular diseases^10^. Recently, the SOMAscan assay was used to develop a 9-protein risk score for cardiovascular outcomes in patients (mean age 67–70 years) with stable coronary heart disease (CHD)^16^. This 9-protein risk score improved the prediction of cardiovascular events (a composite of MI, stroke, hospitalization for HF events) compared to the Framingham score. Ngo *et al*. have used the same technology for the identification of early biomarkers of MI that they have validated by mass spectrometry. They enrolled patients with planned MI who underwent septal ablation for hypertrophic cardiomyopathy^15^. The SOMAscan assay has a larger dynamic range compared to Luminex technology, detecting protein levels at a femtomolar concentration, with a range of eight orders of magnitude^20^. Interestingly, we validated in our population that the BNP measured with the SOMAscan was significantly correlated (r = 0.76, *P* < 0.0001) with the BNP measured at the hospital for low and high levels of plasma BNP (Supplementary Fig. S4).

We used two strategies for analysing the proteins quantified by SOMAscan. Due to more than 200 proteins differentially expressed between the two groups of patients, we first built a molecular network to study the pathophysiological mechanisms underlying HF. Networks are a logical approach for characterizing multidimensional complex interactions, and system biology approaches have been shown to reflect the underlying mechanisms than traditional approaches^7^. The main clusters in the INCA network were associated with the immune system, transcription and translation and signalling pathways. In parallel, we assessed the performance of proteomic models to identify a restricted number of proteins that could predict the early death of HF patients after HF evaluation during hospitalization. By using an adaptive LASSO analysis, we were able to select 6 candidate protein biomarkers that could be divided into 2 subgroups with similar expression in patients who died of CV causes compared to surviving patients: increased MAPK5, MMP1 and MMP7 and decreased C3b, CATS, and F107B.

Three of them, C3, MAPK5 and CATS, had a high centrality in the INCA network, suggesting an important role in HF mechanisms. Indeed, the transcription factor STAT3 by its protective cardiac function, helping maintain metabolic homeostasis, may contribute to myocarditis due to enhanced cardiac IL-6 production and thereby IL-6-induced complement component C3 production^21^. Complement C3 is a central effector pathway of the innate immune system that plays an important role in cardiac remodelling and heart failure. MAPK5 is an intracellular serine/threonine kinase activated by p38MAPKs that has been detected in heart^22^. Recently, it was postulated that it can be a regulator of cardiac fibroblast function by regulating actin cytoskeletal dynamics through phosphorylation of FOXO1 and FOXO3, which are members of the forkhead box family of transcription factors^23^. CATS is a lysosomal cysteine protease that functions in the degradation and turnover of the extracellular matrix. It has been proposed that CATS is involved in TGF-β signalling and myofibroblast differentiation for regulating scar formation in the myocardium after MI in order to preserve LV function^24^. These 3 proteins are involved in pathophysiological processes related to the main clusters identified in the INCA network (immune system, transcription and translation and signalling pathways), showing the utility of system-biological approaches.

We used conventional assays for only 4 of the candidate proteins, C3, CATS, MMP1 and MMP7, because specific assays were not available for MAPK5 and F107B. We confirmed significant modulation for C3, MMP1 and MMP7 and a significant positive correlation with the SOMAscan data for MMP1 (r = 0.842) and MMP7 (r = 0.700). We did not validate CATS, and the discrepancy could be explained by the existence of 2 CATS isoforms that are not distinguished by one of the assays, meaning a lower sensitivity to highlight protein level modulations. We did not observe a significant correlation for C3 as measured by Luminex technology, which does not discriminate between C3a and C3b. In the SOMAscan, specific SOMAmers detect C3a and C3b, but it was also shown that both C3a and C3b have the highest coefficient of variability among the SOMAmers^12^.

We identified 6 candidate proteins that may predict early death in systolic HF patients. Here, we identified that low levels of C3b in HF patients predicted early death. This has already been described for patients with low C3c levels, who had a higher risk of mortality^25^. In contrast, high C3a levels were associated with a higher risk of cardiac events in HF patients^26^. C3 is an innate immune marker that increased following treatment of acute HF, suggesting its involvement in the acute episode^27^. F107B belongs to the FAM107 family of small stress-responsive proteins with functions similar to heat-shock proteins during the cellular stress response^28^. However, until now, F107B function has been poorly investigated due to the lack of tools. Because of their involvement in numerous biological processes, cathepsins, including CATS, have been suggested to be potential circulating biomarkers of HF^29^, but previous studies were conflicting. One prior study in two cohorts of elderly patients showed that higher circulating CATS levels are independently associated with a higher risk of death^30^. Two other studies in chronic HF patients^31^ and in patients with stable coronary heart disease^32^ did not show any difference in plasma CATS level when measured by ELISA, confirming our results.

MMPs are involved in extracellular matrix remodelling during cardiovascular diseases^33,34^. The association between MMP9 and worsening events in chronic HF patients has been described^35^, but there are only few papers about the 2 MMPs identified here. A recent study has shown that MMP1 with protease-activated receptors (PAR) 1 and BNP and NTproBNP were downregulated in obese HF patients^36^. Interestingly, mechanistic research showed that inflammation mediated by MMP1-PAR1 might amplify tumour necrosis factor α signalling in endothelial cells^37^. In addition, myocardial collagen cross-linking, quantified by the serum C-telopeptide for type-I collagen (CITP): MMP1 ratio, is a risk marker of HF hospitalization in patients with hypertensive HF but not with the risk of CV death^38^. CITP was not measured in our proteomic profiling, and we could not evaluate whether patients presenting a phenotype of myocardial fibrosis^39^ were at higher risk of early CV death. MMP7 has been described to promote smooth muscle cell apoptosis by cleaving N-cadherin^40^. MMP7 has been associated with an increased risk of LV remodelling in patients with LV hypertrophy^41^.

Large-scale approaches are becoming increasingly important in the discovery of new biomarkers of many pathologies, especially HF. Indeed, the simultaneous measurement of more than 5000 proteins in plasma is now possible. This can lead to important new perspectives for biomarker discovery. However, these approaches require powerful statistical tools to analyse the large quantity of generated data. It is also interesting to combine both statistical and systems biology approaches to understand the physiopathological pathways involved in HF and to find biomarkers from the network-driven biological levels^7,42^.

In summary, we demonstrated the application of an aptamer-based proteomics platform for the discovery of blood biomarkers associated with the risk of early death in systolic HF patients. Here, we identified 6 proteins (C3, MAPK5, CATS, MMP1, MMP7 and F107B) that could predict early death in HF patients. Some of these candidates were not described as secreted proteins because they do not have a signal peptide (MAPK5 and F107B), targeting them to the endoplasmic reticulum before eventually to be secreted, but we verified their presence in plasma (http://www.peptideatlas.org/). Interestingly, the plasma levels of MAPK5 were associated with the 10-year change in cognitive decline^43^. Their plasma levels were not shown to be associated with age^17^, suggesting that these candidates could also be involved in CV death in elderly HF patients.

Limitations: This is a monocentric case-control study, and our findings needed to be replicates in independent cohorts of HF patients. Our HF patients were mostly men, and our data cannot be extrapolated to women. Although this proteomic platform provides the widest coverage for secreted proteins, coverage of the human proteome remains limited because many analytes are not targeted. It was recently described that the SOMAscan detected 818 secreted proteins among the 2251 proteins described in the human protein atlas, making for a coverage of 36% in our study^42^.

## Materials and Methods

Our institutions (University of Lille, Inserm, “Centre Hospitalier de Lille”, Inria and “Institut Pasteur de Lille”) have approved the study.

All methods were performed as requested by the relevant guidelines and regulations.

The ethics committee of the “Centre Hospitalier de Lille” (CP98/94, 5 November 1998) has approved the INCA study, and written informed consent of each patient has been obtained.

### Study population

All the patients hospitalized for systolic HF (LVEF < 45%) between November 1998 and May 2010 in the “Centre Hospitalier de Lille” were included in the INCA prospective cohort on prognostic indicators. All patients were clinically stable for at least 2 months after inclusion and received optimal medical therapy. At inclusion, patients underwent BNP level assessment, echocardiography and cardiopulmonary testing. A coronary angiogram was also performed to determine the aetiology of LV systolic dysfunction (ischaemic or non-ischaemic). A follow-up was performed at 3 years to assess the clinical outcome. Cardiovascular death corresponded to cardiovascular-related death, urgent transplantation and urgent assist device implantation. Peripheral blood samples were collected at inclusion in tubes containing ethylenediaminetetraacetic acid (EDTA), and plasma samples were stored at −80 °C.

Among all the patients, 168 were selected for inclusion in the proteomic profiling: 84 who died of cardiovascular causes within 3 years (CV death, case) were matched for age, sex and HF aetiology, with 84 patients who were still alive after 3 years (no CV death, control). Continuous variables are presented as mean ± standard deviation (SD) and were compared using Student’s t-test. Categorical variables are expressed as absolute number and/or percentages and were compared using the χ2 test or the Fisher test, as appropriate.

### Proteomic assessment

Proteomic profiling for 1310 proteins was assessed in the plasma of the 168 patients selected using a Slow-Off rate Modified Aptamer (SOMAmers®)-based capture array (version 1.2, SomaLogic, Inc). This technique is based on chemically modified DNA, aptamers called SOMAmers®, which are synthetic oligonucleotides that can bind to ligands^10^. A SOMAmer® reagent is a single-stranded DNA-based aptamer that is chemically modified to enhance binding to conformational protein epitopes with high affinity and specificity. SOMAmers® are coupled with a fluorophore, a photocleavable linker and biotin, and immobilized on beads. The assay measures proteins directly from plasma using a multi-step capture, release, and re-capture enrichment process. First, plasma proteins bind to the bead-immobilized SOMAmers®, and highly specific SOMAmer-protein complexes are formed. SOMAmer®-bound proteins are biotinylated and then released by a photocleavage process. Next, the biotinylated proteins are captured on streptavidin-coupled beads, and SOMAmers are released using a denaturing solution. The fluorophore-tagged modified nucleotides are hybridized with their complementary sequences on a microarray before quantification using an oligo-array plate reader (Agilent Technologies). The resulting fluorescence is quantified and reflects protein levels.

The data were then processed for SOMAscan™ standardization to correct for systematic effects in data introduced during the hybridization process, and 12 hybridization control sequences were introduced into each clinical sample. There was a predetermined global reference RFU for each hybridization control based on independently run assays. A ratio was determined by this global RFU for each control/measured RFU of each hybridization control. The median of the ratios determined the sample-based hybridization scale factor. Each sample was multiplied by its own scale factor.

Median normalization was performed to remove sample or assay biases that may have been due to differences between samples in overall protein concentration, pipetting variation, variation in reagent concentrations, assay timing, and any other source of systematic variability within a single plate run. Each sample was diluted to 40%, 1% or 0.05%. A scale factor was derived for each dilution set, and all the SOMAmers® in each dilution set were scaled together. The median RFU for a SOMAmer® within the sample group was the reference SOMAmer RFU. The ratio of the reference SOMAmer RFU/measured RFU of the SOMAmer in the sample was determined. Within each dilution set, the median SOMAmer ratio was the scale factor for all the SOMAmers in that dilution in that sample. The acceptance criteria for these values were 0.4 to 2.5, based on historic trends in these values^10^.

Two plates were run for this study and four samples (all controls) failed SOMAscan QC criteria and were excluded from the analysis (Supplementary Table S6).

### Data analysis

To identify significantly modulated proteins between patients who died of CV causes after 3 years and the patients who were alive, the Mann-Whitney-Wilcoxon test was performed and adjusted for multiple testing using the Benjamini-Hochberg false discovery rate (FDR)-controlling procedure^44^. Then, as shown in Fig. 1, we used 2 strategies for analysing our high-throughput proteomic profiling.

#### Molecular INCA network building

A molecular network was constructed using the proteins that were significantly modulated in the plasma of INCA patients, i.e., proteins with an adjusted p-value < 0.05 and an absolute log2FC > 0.25. The 203 significantly changed proteins could be mapped to 211 nodes, which were used as “seed nodes” to construct the network model. To build the INCA network, we used the knowledge platform EdgeBox (EdgeLeap’s proprietary knowledge platform), which contained 13 public databases on molecular interactions (Supplementary Table S2): ChBI (http://www.ebi.ac.uk/chebi), ChEMBL (http://www.ebi.ac.uk/chembl), ENCODE (http://encodenets.gersteinlab.org), Ensembl Genes (http://www.ensembl.org), Microcosm (http://www.ebi.ac.uk/enright-srv/microcosm), miRBase (http://www.mirbase.org), miRecords (http://c1.accurascience.com/miRecords), miRTarBase (http://mirtarbase.mbc.nctu.edu.tw), Reactome (http://www.reactome.org), STRING (http://string-db.org), STITCH (http://stitch.embl.de), TFe (http://www.cisreg.ca/cgi-bin/tfe/home.pl), and WikiPathways (www.wikipathways.org). The INCA network was built by including nodes and edges satisfying the following criteria: 1. all molecule nodes that were direct neighbours of at least two seed nodes; 2. all seed nodes that had at least one interaction with another seed node or a node satisfying criterion 1; 3. the edges connected two seed nodes or connected a seed node to a node satisfying criterion 1. “Molecular nodes” refers to genes, miRNAs, metabolites and metabolites (type “protein coding”, “miRNA” or “metabolite”). All the INCA network model visualizations were performed using Cytoscape version 3.2.1^45^.

#### Topological-based cluster analysis

The InfoMap algorithm^46^ as implemented in the igraph R package (version 1.0.1)^47^ was used to perform network clustering (http://igraph.org/r/). The InfoMap algorithm assigned each node in the network to a cluster. Clusters with less than 5 nodes and clusters with only one or fewer edges per node within the cluster were excluded. Then, clusters were annotated to biological pathways by performing overrepresentation analysis on biological pathways using Fisher’s exact test with the Gene Ontology database (http://geneontology.org/) and the Reactome database (version 56). Pathways with fewer than 10 proteins or more than 500 proteins were excluded.

#### Betweenness centrality analysis

For each node in the network, the betweenness centrality was calculated using the igraph R package. The betweenness centrality of a node represents the number of shortest paths between all other nodes in the network that cross the node, normalized by the number of all possible shortest paths. A high betweenness centrality suggests a crucial role of the molecule in the physiopathological process, while a low betweenness centrality indicates a more peripheral role.

#### Penalized regression analysis

To select a few proteins that could explain the early death of patients, a framework based on penalized logistic regressions was used. Our study sample size was mainly determined by the size of the initial cohort and the proteomic profiling assay performed. However, approaches able to deal with the high dimensional setting such as Ridge^48^ and LASSO^49^ penalties were chosen to take into account the high number of variables compared to the available number of individuals and the potential problems of correlation between the proteins quantified. Such approaches enjoy consistency properties when the number of significant variables is small compared with the total number of variables, thus making it possible to perform relevant variables selection even for moderated sample size. First, a ridge regression was applied to explain the early death of HF patients with the 203 proteins. Second, the resulting coefficients were inversed to be used as weights in the adaptive LASSO^50^, thus performing variable selection. All regularization parameters were tuned by leave-one-out cross-validation. To limit the influence of potential extreme individuals, 168 adaptive LASSOs were performed with the pre-defined regularization parameters and weights calculated above, each time removing one individual among the 168. We only kept the proteins whose frequency of selection by the adaptive LASSO was higher than 0.90 among the 168 different training subsets. The entire procedure was implemented in R, using the R package glmnet (version 2.0.16) for penalized regressions^51^.

### CATS, MMP1, MMP7 and C3 measurements

CATS was quantified using the human cathepsin S ELISA (Abcam ab155427) according to the manufacturer’s instructions. Plasma samples were diluted 1/1000, and CATS concentration was determined by calculating the mean absorbance for each standard and sample and subtracting the average zero standard optical density.

Plasma levels of MMP1, MMP7 and C3 were measured using Luminex technology (R&D systems FCSTM07-02 for MMP1 and MMP7 and Merck-Millipore HCMP2MAG-19K-07 for C3), according to the manufacturer’s instructions. This technique relies on the use of beads that have different colour codes depending on the targeted analyte. Beads are coated with analyte-specific antibodies. First, plasma samples were added to a mixture of beads pre-coated with analyte-specific antibodies, and proteins were captured. Biotinylated antibodies specific to the analytes of interest were added and formed complexes with the analyte-specific antibodies. Phycoerythrin (PE)-conjugated streptavidin was added and bound to the biotinylated antibodies. Beads were read using 2 lasers: one that detected the analyte and the second that detected PE, which reflected the analyte concentration. All samples were analysed with the Bio-Plex system (Bio-Rad Laboratories, Hercules, CA) following the manufacturer’s instructions. For MMP1 and MMP7, plasma samples were diluted 1/4 and for C3, 1/40000. The detection limit was 0.3–1.1 pg/mL for MMP1, 1.3–6.6 pg/mL for MMP7 and 0.08 ng/mL for C3. Experimental data were analysed by fitting a four-parameter logistic curve to the standard analyte curves, except for MMP7.

Materials, data and associated protocols are promptly available to readers without undue qualifications in material transfer agreements upon request.

## Supplementary information

supplementary informations

## Data Availability

The datasets generated during and/or analysed during the current study are available from the corresponding author on reasonable request.
