# Supplementary material for: Circulating proteomic signature of early death in heart failure patients with reduced ejection fraction
Source: Sci Rep. 2019 Dec 16;9:19202. doi: 10.1038/s41598-019-55727-1 (PMC6914779; doi:10.1038/s41598-019-55727-1)
Supplement: Supplementary file 1 — supplementary informations [file 41598_2019_55727_MOESM1_ESM.pdf]

SREP-19-23335A-R3

SUPPLEMENTARY DATA

Circulating proteomic signature of early death in heart failure patients with reduced ejection fraction

Marie Cuvelliez[1,2]*, Vincent Vandewalle[3,4]*, Maxime Brunin[4], Olivia Beseme[1,2], Audrey Hulot[4], Pascal de Groote[1,3,5], Philippe Amouyel[1,2,6], Christophe Bauters[1,2,6]#, Guillemette Marot[3,4]#, Florence Pinet[1,2]#‡

SUPPLEMENTARY FIGURES

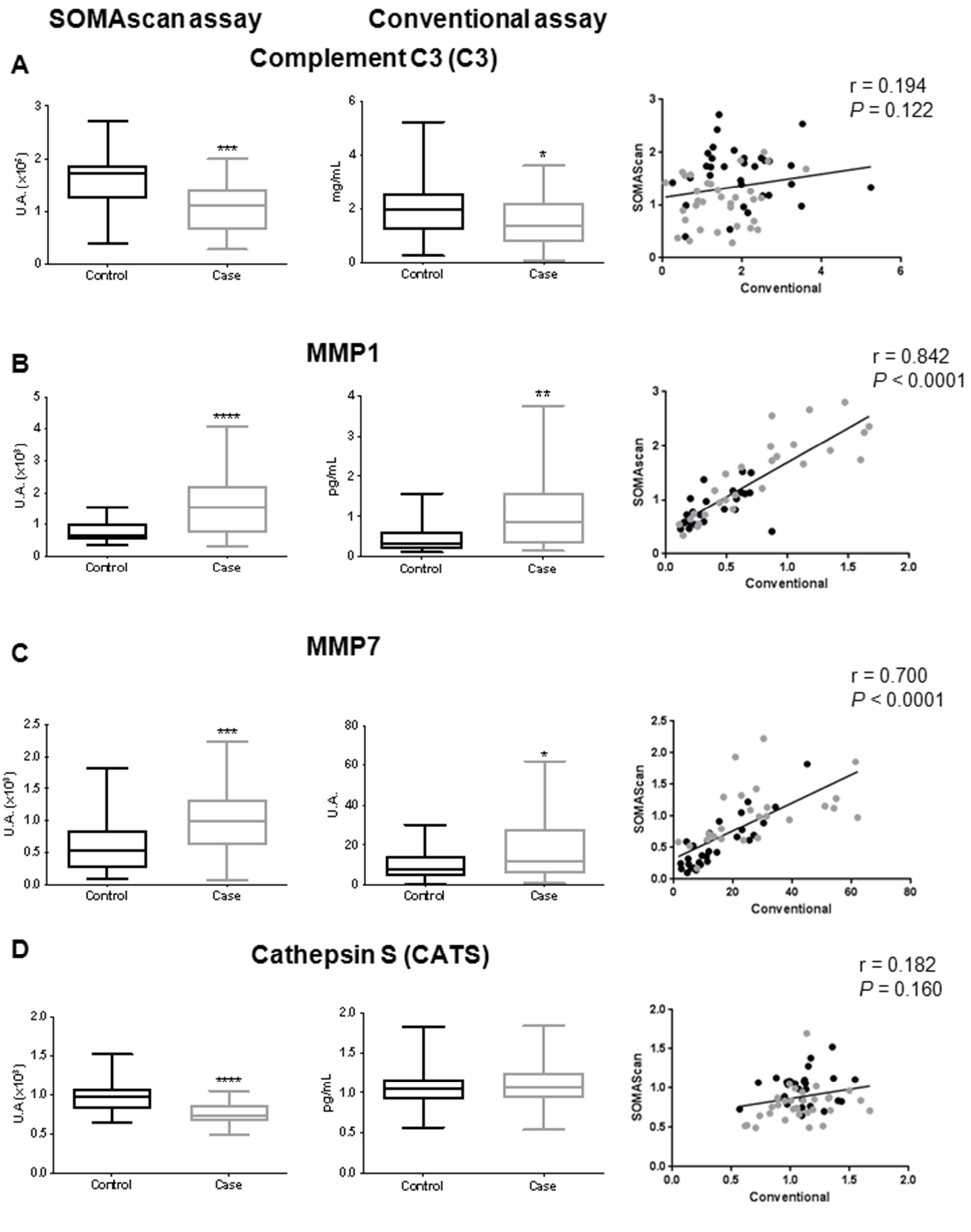

Supplementary Figure S1 online. Correlation for the 4 selected proteins quantified by Somascan and conventional assays. Quantification of complement C3 (**a**), MMP1 (**b**), MMP7 (**c**) and cathepsin S

(**d**) by SOMAscan assay (left panels) and conventional assays (middle panels) were performed in the same patients (33 who died of CV causes and 33 alive after 3 years) of the INCA study (Table 1). Data are expressed in arbitrary units (A.U) corresponding to relative fluorescent units (RFU) for the SOMASscan assay and to concentration for the conventional assays, except for MMP7. Data are presented as box-and-whisker plots showing median (line) and min to max (whisker). Statistical significance was determined by the Wilcoxon-Mann-Whitney test. **P*<0.05, ***P*<0.01, ****P*<0.001, *****P*<0.0001. The 4 proteins quantified by SOMAscan and conventional assays were tested for correlation (right panels). Data are presented as individual value for each patient in the two groups, with Case (patients who died after 3 years of follow-up) in grey colour and Control (patients alive after 3 years of follow-up) in black colour. The Spearman's rank correlation coefficient (r) and the *P* value are indicated on the graph.

```
P25774|CATS_HUMAN   MKRLVCVLLVCSSAVAQLHKDPTLDHHWHLWKKTYGKQYKEKNEEAVRRLIWEKNLKFVM 60
P25774-2|CATS_HUMAN MKRLVCVLLVCSSAVAQLHKDPTLDHHWHLWKKTYGKQYKEKNEEAVRRLIWEKNLKFVM 60
                    ************************************************************

P25774|CATS_HUMAN   LHNLEHSMGMHSYDLGMNHLGDMTSEEVMSLMSSLRVPSQWQRNITYKSNPNRILPDSVD 120
P25774-2|CATS_HUMAN LHNLEHSMGMHSYDLGMNHLGDM------------------------------------- 83
                    ***********************

P25774|CATS_HUMAN   WREKGCVTEVKYQGSCGACWAFSAVGALEAQLKLKTGKLVSLSAQNLVDCSTEKYGNKGC 180
P25774-2|CATS_HUMAN -------------GSCGACWAFSAVGALEAQLKLKTGKLVSLSAQNLVDCSTEKYGNKGC 130
                                 ***********************************************

P25774|CATS_HUMAN   NGGFMTTAFQYIIDNKGIDSDASYPYKAMDQKCQYDSKYRAATCSKYTELPYGREDVLKE 240
P25774-2|CATS_HUMAN NGGFMTTAFQYIIDNKGIDSDASYPYKAMDQKCQYDSKYRAATCSKYTELPYGREDVLKE 190
                    ************************************************************

P25774|CATS_HUMAN   AVANKGPVSVGVDARHPSFFLYRSGVYYEPSCTQNVNHGVLVVGYGDLNGKEYWLVKNSW 300
P25774-2|CATS_HUMAN AVANKGPVSVGVDARHPSFFLYRSGVYYEPSCTQNVNHGVLVVGYGDLNGKEYWLVKNSW 250
                    ************************************************************

P25774|CATS_HUMAN   GHNFGEEGYIRMARNKGNHCGIASFPSYPEI 331
P25774-2|CATS_HUMAN GHNFGEEGYIRMARNKGNHCGIASFPSYPEI 281
                    *******************************
```

Supplementary Figure S2 online. Sequence alignment of the 2 human isoforms of Cathepsin S (CATS). The alignment was performed with the Align tool from UniProtKB database (https://www.uniprot.org/align/) and showed the 50 AA (from 184 to 133) highlighted in grey which are deleted in the isoform 2. The identity between the 2 isoforms is 84.894%.

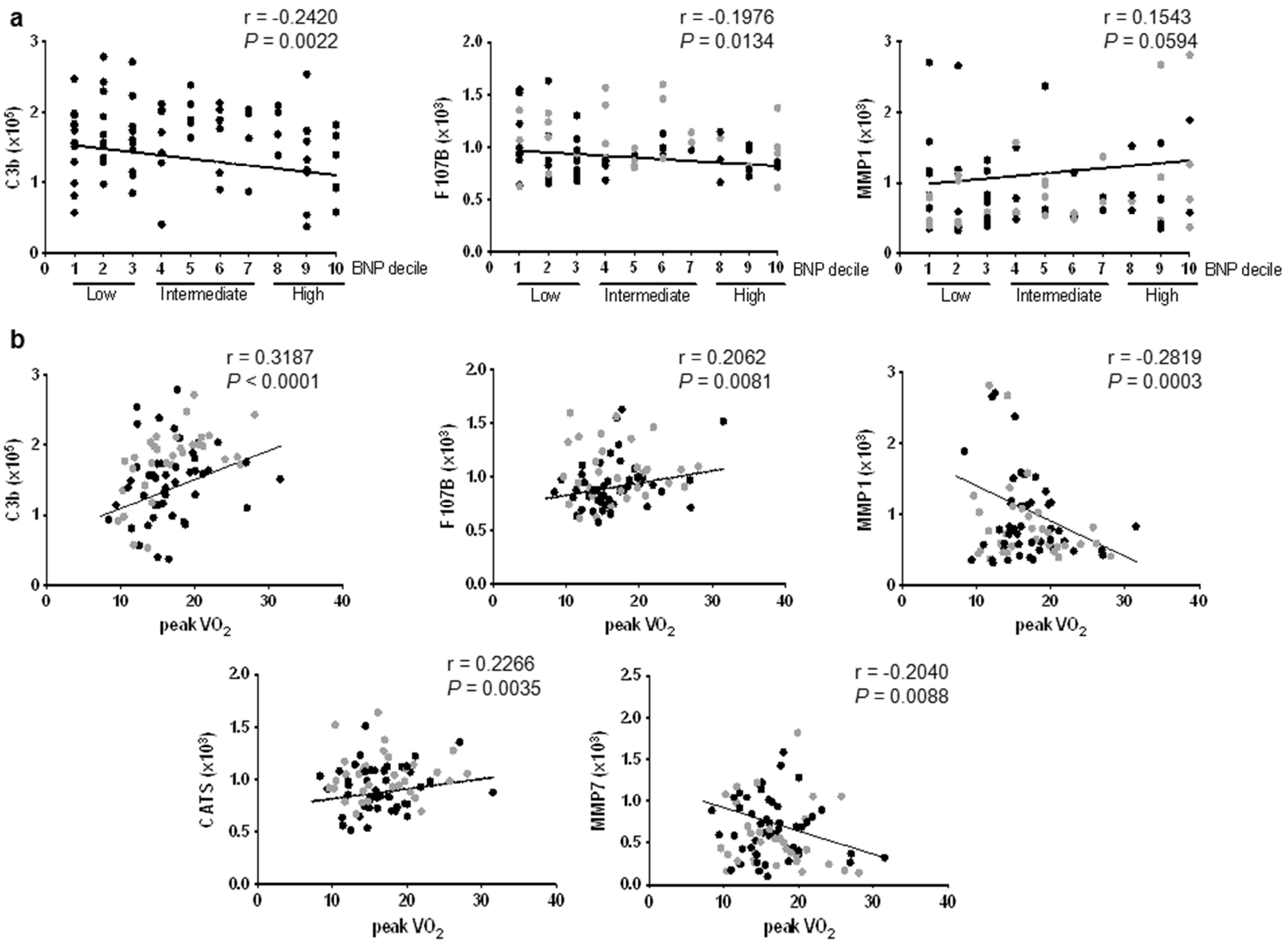

a
r = -0.2420
P = 0.0022
C3b (x10^5)
BNP decile
Low
Intermediate
High
r = -0.1976
P = 0.0134
F107B (x10^3)
r = 0.1543
P = 0.0594
MMP1 (x10^3)
b
r = 0.3187
P < 0.0001
peak VO2
r = 0.2062
P = 0.0081
r = -0.2819
P = 0.0003
r = 0.2266
P = 0.0035
CATS (x10^3)
r = -0.2040
P = 0.0088
MMP7 (x10^3)

Supplementary Figure S3 online. Correlation of BNP and peak VO_2 values with the 6 protein candidates. Only the statistically significant correlations are shown. **a**: Correlation of BNP with C3b (left panel), F107B (middle panel) and MMP1 (right panel). The BNP level quantified at the hospital was categorized as low (deciles 1, 2 and 3), intermediate (deciles 4, 5, 6 and 7) or high (deciles 8, 9 and 10) for each individual patient. **b:** Correlation of peak VO_2 with C3b (top left panel), F107B (top middle panel), MMP1 (top right panel), CATS (bottom left panel) and MMP7 (bottom right panel). Data are presented as individual value for each patient in the two groups, with Case (patients who died after 3 years of follow-up) in grey colour and Control (patients alive after 3 years of follow-up) in black colour. The Spearman's rank correlation coefficient (r) and the *P* value were calculated and indicated on the graph.

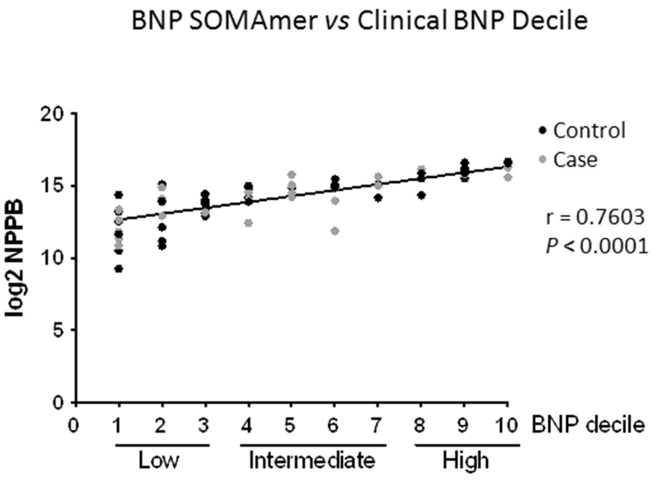

Supplementary Figure S4 online. Correlation of BNP values in INCA population. BNP was quantified in the plasma of HF patients respectively, at the hospital and by SOMAscan assay. The BNP level quantified at the hospital was categorized as low (deciles 1, 2 and 3), intermediate (deciles 4, 5, 6 and 7) or high (deciles 8, 9 and 10) for each individual patient. Data of the BNP quantified by SOMAscan were log2 transformed. Data are presented as individual value for each patient in the two groups, with Case (patients who died after 3 years of follow-up) in grey colour and Control (patients alive after 3 years of follow-up) in black colour. The Spearman's rank correlation coefficient (r) and the *P* value were calculated and indicated on the graph.

Supplementary Table S1 online. List of the 203 proteins differentially expressed between the patients who died (Case, n=84) and the patients alive (Control, n=80) after 3 years follow-up.

Protein symbol	Protein name	UniProt ID*	mean Control	mean Case	SD Control	SD Case	log2 ratio	M-W p-value	M-W adjusted p-value
RAN	GTP-binding nuclear protein Ran	P62826	9.212	10.495	1.860	1.757	-1.283	0.00001	0.00055
SRCN1	Proto-oncogene tyrosine-protein kinase Src	P12931	11.895	13.078	1.827	2.094	-1.183	0.00010	0.00213
PAK6	Serine/threonine-protein kinase PAK 6	Q9NQU5	11.617	12.639	1.497	1.667	-1.022	0.00006	0.00168
BTK	Tyrosine-protein kinase BTK	Q06187	11.077	12.004	1.569	1.794	-0.927	0.00046	0.00452
IF4G2	Eukaryotic translation initiation factor 4 gamma 2	P78344	10.103	11.028	1.518	1.466	-0.925	0.00020	0.00291
PKC-A	Protein kinase C alpha type	P17252	12.699	13.597	1.606	1.647	-0.897	0.00062	0.00541
PF-4	Platelet factor 4	P02776	14.852	15.730	1.615	1.655	-0.878	0.00019	0.00291
Tropomyosin 4	Tropomyosin alpha-4 chain	P67936	13.897	14.770	1.762	1.653	-0.873	0.00148	0.00961
SGTA	Small glutamine-rich tetratricopeptide repeat-containing protein alpha	O43765	10.850	11.702	0.951	1.043	-0.853	0.00000	0.00007
Cyclophilin F	Peptidyl-prolyl cis-trans isomerase F, mitochondrial	P30405	12.859	13.707	1.903	1.751	-0.848	0.00514	0.02209
Alpha enolase	Alpha-enolase	P06733	12.684	13.521	2.168	1.893	-0.836	0.00967	0.03609

CSK	Tyrosine-protein kinase CSK	P41240	11.236	12.066	1.604	1.753	-0.830	0.00169	0.01029
NCC27	Chloride intracellular channel protein 1	O00299	10.912	11.735	1.160	1.500	-0.823	0.00043	0.00426
GSK-3 alpha/beta	Glycogen synthase kinase-3 alpha/beta	P49840 P49841	11.681	12.477	1.265	1.358	-0.797	0.00015	0.00262
M2-PK	Pyruvate kinase PKM	P14618	11.369	12.151	1.476	1.620	-0.782	0.00166	0.01021
RANTES	C-C motif chemokine 5	P13501	14.097	14.875	1.323	1.164	-0.778	0.00011	0.00216
PPID	Peptidyl-prolyl cis-trans isomerase D	Q08752	10.367	11.145	1.658	1.785	-0.778	0.00377	0.01810
MMP-1	Interstitial collagenase	P03956	9.612	10.386	0.803	0.897	-0.773	0.00000	0.00005
eIF-4H	Eukaryotic translation initiation factor 4H	Q15056	12.315	13.079	1.554	1.317	-0.764	0.00141	0.00937
RAC1	Ras-related C3 botulinum toxin substrate 1	P63000	13.493	14.245	1.286	1.263	-0.753	0.00019	0.00291
Carbonic anhydrase 13	Carbonic anhydrase 13	Q8N1Q1	9.804	10.556	1.233	1.393	-0.752	0.00049	0.00463
PKC-B-II	Protein kinase C beta type (splice variant beta-II)	P05771	10.308	11.047	1.618	1.720	-0.738	0.00536	0.02293
Sorting nexin 4	Sorting nexin-4	O95219	11.824	12.561	1.153	1.207	-0.737	0.00014	0.00255
RAC3	Ras-related C3 botulinum toxin substrate 3	P60763	11.530	12.260	0.885	1.060	-0.730	0.00001	0.00055
IMB1	Importin subunit beta-1	Q14974	12.342	13.061	1.131	1.048	-0.719	0.00002	0.00074

NAP-2	Neutrophil-activating peptide 2	P02775	12.313	13.018	1.255	1.266	-0.705	0.00020	0.00291
Sphingosine kinase 1	Sphingosine kinase 1	Q9NYA1	10.629	11.327	1.236	1.399	-0.697	0.00139	0.00931
Histone H1.2	Histone H1.2	P16403	8.630	9.326	1.233	1.407	-0.696	0.00083	0.00656
CTAP-III	Connective tissue-activating peptide III	P02775	13.631	14.321	1.248	1.242	-0.690	0.00024	0.00323
SBDS	Ribosome maturation protein SBDS	Q9Y3A5	11.294	11.974	0.961	1.148	-0.679	0.00011	0.00213
Olfactomedin-4	Olfactomedin-4	Q6UX06	8.154	8.832	1.009	1.802	-0.678	0.00014	0.00248
Thrombospondin-1	Thrombospondin-1	P07996	11.542	12.219	1.264	1.233	-0.677	0.00074	0.00615
PPIE	Peptidyl-prolyl cis-trans isomerase E	Q9UNP9	9.580	10.252	0.840	0.954	-0.672	0.00001	0.00055
LYN	Tyrosine-protein kinase Lyn	P07948	10.716	11.387	1.403	1.396	-0.671	0.00228	0.01260
XPNPEP1	Xaa-Pro aminopeptidase 1	Q9NQW7	13.150	13.808	1.006	1.003	-0.658	0.00005	0.00161
NDP kinase B	Nucleoside diphosphate kinase B	P22392	12.991	13.647	1.223	1.264	-0.656	0.00049	0.00463
Aflatoxin B1 aldehyde reductase	Aflatoxin B1 aldehyde reductase member 2	O43488	9.313	9.967	1.293	1.424	-0.654	0.00364	0.01771
SP-D	Pulmonary surfactant-associated protein D	P35247	13.446	14.099	1.774	1.746	-0.653	0.00494	0.02156
Flt-3	Receptor-type tyrosine-protein	P36888	10.426	11.076	0.931	1.113	-0.651	0.00012	0.00227

	kinase FLT3								
PRKACA	cAMP-dependent protein kinase catalytic subunit alpha	P17612	12.374	13.017	1.504	1.349	-0.643	0.00716	0.02833
37694	14-3-3 protein family	P31946, P62258, P61981, Q04917, P27348, P63104, P31947	10.561	11.197	1.130	1.233	-0.637	0.00085	0.00663
LYNB	Tyrosine-protein kinase Lyn, isoform B	P07948	12.992	13.618	1.407	1.344	-0.626	0.00446	0.02026
BARK1	beta-adrenergic receptor kinase 1	P25098	11.949	12.570	1.394	1.332	-0.621	0.00332	0.01637
CK2-A1:B	Casein kinase II 2-alpha:2-beta heterotetramer	P68400 P67870	12.653	13.273	1.067	0.917	-0.620	0.00008	0.00196
MMP-7	Matrilysin	P09237	9.044	9.664	0.926	0.962	-0.620	0.00003	0.00112
GPVI	Platelet glycoprotein VI	Q9HCN6	12.357	12.960	0.878	0.949	-0.604	0.00010	0.00210
Esterase D	S-formylglutathione hydrolase	P10768	12.133	12.720	0.897	0.900	-0.588	0.00002	0.00072
PA2G4	Proliferation-associated protein 2G4	Q9UQ80	9.937	10.523	1.100	1.202	-0.586	0.00057	0.00510
HSP 90a/b	Heat shock protein HSP 90-alpha/beta	P07900 P08238	12.478	13.062	0.876	0.967	-0.584	0.00007	0.00185
TCTP	Translationally-controlled tumor protein	P13693	12.542	13.112	1.028	1.065	-0.570	0.00075	0.00619
PPAC	Low molecular	P24666	10.891	11.459	1.047	1.143	-0.568	0.00200	0.01155

	weight phosphotyrosine protein phosphatase								
SNAA	Alpha-soluble NSF attachment protein	P54920	11.868	12.432	0.911	1.004	-0.564	0.00030	0.00363
41	Protein 4.1	P11171	13.562	14.125	1.081	0.936	-0.564	0.00026	0.00339
SMAD2	Mothers against decapentaplegic homolog 2	Q15796	11.375	11.937	1.075	1.126	-0.563	0.00187	0.01110
Caspase-3	Caspase-3	P42574	12.016	12.572	1.191	1.084	-0.556	0.00113	0.00811
Myokinase, human	Adenylate kinase isoenzyme 1	P00568	13.448	14.002	1.086	1.055	-0.553	0.00002	0.00081
CPNE1	Copine-1	Q99829	10.170	10.716	1.445	1.421	-0.547	0.00995	0.03662
Calpain I	Calpain I	P07384 P04632	14.327	14.862	0.863	0.976	-0.534	0.00022	0.00310
GCP-2	C-X-C motif chemokine 6	P80162	9.528	10.057	0.709	0.681	-0.529	0.00001	0.00055
PLPP	Pyridoxal phosphate phosphatase	Q96GD0	11.038	11.567	0.855	0.808	-0.529	0.00009	0.00209
AMPM2	Methionine aminopeptidase 2	P50579	10.769	11.297	0.903	1.046	-0.528	0.00070	0.00594
CHIP	E3 ubiquitin-protein ligase CHIP	Q9UNE7	10.807	11.320	0.878	0.918	-0.513	0.00020	0.00294
CRK	Adapter molecule crk	P46108	12.564	13.067	0.766	0.624	-0.503	0.00001	0.00055
NSF1C	NSFL1 cofactor p47	Q9UNZ2	8.868	9.370	0.864	0.637	-0.502	0.00002	0.00081
Stress-induced-phosphoprotein 1	Stress-induced-phosphoprotein 1	P31948	11.925	12.426	0.854	0.922	-0.502	0.00058	0.00519

RPS6KA3	Ribosomal protein S6 kinase alpha-3	P51812	9.363	9.864	1.013	0.917	-0.501	0.00036	0.00401
AREG	Amphiregulin	P15514	10.698	11.198	0.853	0.813	-0.499	0.00015	0.00262
STAT1	Signal transducer and activator of transcription 1-alpha/beta	P42224	11.919	12.414	1.191	1.042	-0.495	0.00381	0.01819
IL-2 sRg	Cytokine receptor common subunit gamma	P31785	10.676	11.170	0.787	0.772	-0.494	0.00007	0.00189
FER	Tyrosine-protein kinase Fer	P16591	7.513	8.005	1.110	1.127	-0.493	0.00371	0.01788
14-3-3 protein beta/alpha	14-3-3 protein beta/alpha	P31946	13.918	14.410	1.164	1.124	-0.492	0.00709	0.02814
VAV	Proto-oncogene vav	P15498	10.861	11.349	1.409	1.375	-0.488	0.01014	0.03701
14-3-3 protein zeta/delta	14-3-3 protein zeta/delta	P63104	16.122	16.609	1.049	0.948	-0.487	0.00309	0.01548
DRG-1	Vacuolar protein sorting-associated protein VTA1 homolog	Q9NP79	13.659	14.143	1.092	1.070	-0.484	0.00233	0.01271
HSP 60	60 kDa heat shock protein, mitochondrial	P10809	12.233	12.717	0.900	0.956	-0.484	0.00059	0.00522
NSE	Gamma-enolase	P09104	14.496	14.973	0.750	0.677	-0.476	0.00001	0.00055
STAT3	Signal transducer and activator of transcription 3	P40763	9.236	9.713	1.046	1.081	-0.476	0.00247	0.01307
SARP-2	Secreted frizzled-related protein 1	Q8N474	11.555	12.030	1.111	1.271	-0.475	0.00569	0.02374
BAD	Bcl2-associated agonist of cell	Q92934	12.313	12.785	0.856	0.856	-0.472	0.00070	0.00594

	death								
Peroxiredoxin-6	Peroxiredoxin-6	P30041	8.381	8.850	0.810	0.959	-0.469	0.00095	0.00708
IMDH1	Inosine-5'-monophosphate dehydrogenase 1	P20839	11.029	11.482	1.421	1.198	-0.452	0.00037	0.00403
FGF-16	Fibroblast growth factor 16	O43320	10.324	10.775	0.745	0.719	-0.451	0.00009	0.00203
Gro-b/g	Gro-beta/gamma	P19876 P19875	8.111	8.559	0.737	0.705	-0.448	0.00009	0.00209
DEAD-box protein 19B	ATP-dependent RNA helicase DDX19B	Q9UMR2	11.122	11.567	0.939	0.943	-0.445	0.00243	0.01296
amyloid precursor protein	Amyloid beta A4 protein	P05067	14.858	15.301	0.878	0.890	-0.443	0.00121	0.00837
HSP 90b	Heat shock protein HSP 90-beta	P08238	14.684	15.121	0.698	0.716	-0.437	0.00009	0.00209
Tpo	Thrombopoietin	P40225	7.462	7.898	0.676	0.796	-0.436	0.00012	0.00239
METAP1	Methionine aminopeptidase 1	P53582	8.630	9.060	0.909	0.965	-0.430	0.00360	0.01765
MAPK2	MAP kinase-activated protein kinase 2	P49137	10.521	10.950	1.084	1.057	-0.429	0.00486	0.02156
YES	Tyrosine-protein kinase Yes	P07947	9.347	9.775	0.528	0.703	-0.428	0.00001	0.00064
I-TAC	C-X-C motif chemokine 11	O14625	11.775	12.202	0.680	1.050	-0.427	0.00389	0.01834
HMG-1	High mobility group protein B1	P09429	11.940	12.353	1.257	1.139	-0.412	0.00494	0.02156
UBE2N	Ubiquitin-conjugating enzyme E2 N	P61088	12.084	12.494	0.947	0.928	-0.410	0.00489	0.02156
Midkine	Midkine	P21741	13.325	13.734	1.145	0.912	-0.409	0.00024	0.00325

Triosephosphate isomerase	Triosephosphate isomerase	P60174	12.199	12.608	0.936	0.919	-0.409	0.00450	0.02040
NMT1	Glycylpeptide N-tetradecanoyltransferase 1	P30419	10.897	11.302	0.847	0.948	-0.405	0.00752	0.02958
EF-1-beta	Elongation factor 1-beta	P24534	9.079	9.483	0.545	0.694	-0.404	0.00006	0.00174
PDE5A	cGMP-specific 3',5'-cyclic phosphodiesterase	O76074	11.521	11.917	0.881	0.943	-0.396	0.00514	0.02209
Adiponectin	Adiponectin	Q15848	10.830	11.223	0.763	0.793	-0.393	0.00283	0.01455
PDGF-AA	Platelet-derived growth factor subunit A	P04085	13.298	13.684	0.953	0.966	-0.386	0.00151	0.00969
LDH-H 1	L-lactate dehydrogenase B chain	P07195	9.564	9.947	0.513	0.543	-0.383	0.00001	0.00055
eIF-5A-1	Eukaryotic translation initiation factor 5A-1	P63241	14.515	14.897	0.777	0.708	-0.383	0.00123	0.00843
FGF-8A	Fibroblast growth factor 8 isoform A	P55075	8.044	8.423	0.626	0.646	-0.378	0.00018	0.00291
UB2L3	Ubiquitin-conjugating enzyme E2 L3	P68036	10.032	10.403	0.866	0.750	-0.371	0.00090	0.00679
Peroxiredoxin-1	Peroxiredoxin-1	Q06830	10.873	11.243	0.705	0.650	-0.370	0.00036	0.00400
JAM-C	Junctional adhesion molecule C	Q9BX67	11.602	11.971	0.538	0.573	-0.369	0.00002	0.00075
FGF-6	Fibroblast growth factor 6	P10767	8.190	8.554	0.516	0.638	-0.365	0.00020	0.00291
ERAB	3-hydroxyacyl-	Q99714	9.721	10.085	0.593	0.728	-0.365	0.00030	0.00363

	CoA dehydrogenase type-2								
DKK1	Dickkopf-related protein 1	O94907	14.436	14.798	0.823	0.875	-0.362	0.00181	0.01086
MK08	Mitogen-activated protein kinase 8	P45983	9.334	9.694	0.979	0.878	-0.360	0.00611	0.02516
α-Synuclein	Alpha-synuclein	P37840	12.612	12.972	0.864	0.915	-0.360	0.00086	0.00663
MAPK14	Mitogen-activated protein kinase 14	Q16539	10.175	10.532	0.840	0.915	-0.356	0.01005	0.03686
PPase	Inorganic pyrophosphatase	Q15181	14.197	14.552	0.683	0.640	-0.354	0.00136	0.00924
NACA	Nascent polypeptide-associated complex subunit alpha	Q13765	12.416	12.768	0.576	0.523	-0.352	0.00001	0.00055
SMAD3	Mothers against decapentaplegic homolog 3	P84022	12.452	12.798	0.777	0.813	-0.346	0.00202	0.01155
CaMKK alpha	Calcium/calmodulin-dependent protein kinase kinase 1	Q8N5S9	7.988	8.331	0.595	0.645	-0.343	0.00052	0.00482
RS3A	40S ribosomal protein S3a	P61247	8.911	9.253	0.739	0.710	-0.341	0.00007	0.00190
PSA6	Proteasome subunit alpha type-6	P60900	9.296	9.636	0.554	0.539	-0.340	0.00006	0.00168
Transketolase	Transketolase	P29401	15.026	15.363	0.835	0.797	-0.336	0.00913	0.03457
KREM2	Kremen protein 2	Q8NCW0	10.778	11.113	0.657	0.601	-0.334	0.00095	0.00708
PDGF-BB	Platelet-derived growth factor	P01127	14.784	15.119	1.155	1.141	-0.334	0.01368	0.04653

	subunit B								
SSRP1	FACT complex subunit SSRP1	Q08945	8.732	9.064	1.109	0.900	-0.333	0.00066	0.00570
UCRP	Ubiquitin-like protein ISG15	P05161	10.930	11.259	0.678	0.671	-0.329	0.00161	0.01006
HEMK2	HemK methyltransferase family member 2	Q9Y5N5	10.797	11.125	0.652	0.750	-0.328	0.00189	0.01110
Cathepsin A	Lysosomal protective protein	P10619	12.527	12.849	0.677	0.636	-0.322	0.00202	0.01155
RBM39	RNA-binding protein 39	Q14498	9.025	9.345	1.022	0.987	-0.321	0.00042	0.00419
SE6L2	Seizure 6-like protein 2	Q6UXD5	10.314	10.634	0.764	0.642	-0.320	0.00045	0.00438
BCL2-like 1 protein	Bcl-2-like protein 1	Q07817	9.435	9.749	0.589	0.686	-0.314	0.00299	0.01511
IL-3 Ra	Interleukin-3 receptor subunit alpha	P26951	9.584	9.897	0.686	0.663	-0.314	0.00071	0.00601
RS7	40S ribosomal protein S7	P62081	10.356	10.668	1.322	1.042	-0.312	0.00150	0.00968
AMPK a2b2g1	AMP Kinase (alpha2beta2gamma1)	P54646 O43741 P54619	10.751	11.061	0.945	0.804	-0.311	0.01190	0.04180
DHH	Desert hedgehog protein N-product	O43323	9.269	9.576	0.489	0.519	-0.307	0.00008	0.00201
Rab GDP dissociation inhibitor beta	Rab GDP dissociation inhibitor beta	P50395	13.627	13.933	0.737	0.661	-0.306	0.00213	0.01191
ING1	Inhibitor of growth protein 1	Q9UK53	13.101	13.405	0.881	0.872	-0.304	0.01485	0.04975
ARI3A	AT-rich interactive domain-containing protein 3A	Q99856	8.615	8.917	0.854	0.828	-0.302	0.00110	0.00801

KPCI	Protein kinase C iota type	P41743	9.746	10.047	0.672	0.652	-0.300	0.00120	0.00837
Ubiquitin	Ubiquitin	P62979	10.153	10.452	0.581	0.818	-0.298	0.00387	0.01831
RSPO4	R-spondin-4	Q2I0M5	8.303	8.599	0.568	0.696	-0.295	0.00443	0.02023
Chymase	Chymase	P23946	9.038	9.331	0.504	0.534	-0.293	0.00000	0.00051
P-Selectin	P-selectin	P16109	13.931	14.222	0.386	0.494	-0.291	0.00024	0.00323
prostatic binding protein	Phosphatidylethanolamine-binding protein 1	P30086	11.882	12.169	0.620	0.623	-0.287	0.00117	0.00831
CK2-A2:B	Casein kinase II 2-alpha':2-beta heterotetramer	P19784 P67870	8.230	8.515	0.826	0.786	-0.285	0.01109	0.03939
EPI	Epiregulin	O14944	9.922	10.206	0.507	0.771	-0.285	0.00139	0.00931
EDAR	Tumor necrosis factor receptor superfamily member EDAR	Q9UNE0	9.441	9.725	0.495	0.597	-0.284	0.00107	0.00782
Protein disulfide isomerase A3	Protein disulfide-isomerase A3	P30101	11.786	12.069	0.526	0.665	-0.283	0.00397	0.01859
hnRNP A/B	Heterogeneous nuclear ribonucleoprotein A/B	Q99729	9.743	10.026	1.347	1.037	-0.283	0.00441	0.02023
IL-8	Interleukin-8	P10145	9.214	9.495	0.595	0.639	-0.281	0.00060	0.00528
Ubiquitin+1	Ubiquitin+1, truncated mutation for UbB	P62979	11.001	11.280	0.792	0.664	-0.279	0.00165	0.01020
PDE11	Dual 3',5'-cyclic-AMP and -GMP phosphodiesterase 11A	Q9HCR9	9.909	10.184	0.563	0.817	-0.275	0.01224	0.04276
ANP	Atrial natriuretic factor	P01160	9.513	9.787	0.507	0.546	-0.274	0.00042	0.00419

CDC37	Hsp90 co-chaperone Cdc37	Q16543	8.019	8.291	0.419	0.558	-0.272	0.00041	0.00419
PKC-Z	Protein kinase C zeta type	Q05513	9.470	9.740	0.651	0.597	-0.270	0.00007	0.00185
FGF7	Fibroblast growth factor 7	P21781	10.313	10.582	0.438	0.370	-0.269	0.00000	0.00006
Protease nexin I	Glia-derived nexin	P07093	10.406	10.674	0.645	0.637	-0.268	0.00599	0.02481
Cytochrome P450 3A4	Cytochrome P450 3A4	P08684	13.752	14.019	0.507	0.611	-0.268	0.00484	0.02155
ASM3A	Acid sphingomyelinase-like phosphodiesterase 3a	Q92484	12.345	12.611	0.486	0.528	-0.266	0.00014	0.00248
MDHC	Malate dehydrogenase, cytoplasmic	P40925	14.107	14.373	0.589	0.574	-0.266	0.00292	0.01485
NUDC3	NudC domain-containing protein 3	Q8IVD9	8.130	8.395	0.501	0.728	-0.265	0.00038	0.00407
IDE	Insulin-degrading enzyme	P14735	10.397	10.662	0.489	0.485	-0.264	0.00047	0.00459
Dkk-4	Dickkopf-related protein 4	Q9UBT3	12.428	12.691	0.580	0.625	-0.264	0.00322	0.01597
UFM1	Ubiquitin-fold modifier 1	P61960	10.452	10.712	0.649	0.610	-0.259	0.00829	0.03222
Gro-a	Growth-regulated alpha protein	P09341	10.999	11.257	0.629	0.545	-0.258	0.00036	0.00400
PSD7	26S proteasome non-ATPase regulatory subunit 7	P51665	8.497	8.754	0.444	0.371	-0.257	0.00001	0.00055
RSK-like protein kinase	Ribosomal protein S6 kinase alpha-5	O75582	7.249	7.506	0.506	0.595	-0.256	0.00138	0.00930

Bcl-2	Apoptosis regulator Bcl-2	P10415	10.013	10.267	0.789	0.514	-0.254	0.00010	0.00213
MAPK5	MAP kinase-activated protein kinase 5	Q8IW41	8.749	9.000	0.437	0.553	-0.251	0.00085	0.00663
Calpastatin	Calpastatin	P20810	13.891	13.637	0.487	0.468	0.253	0.00013	0.00248
CD97	CD97 antigen	P48960	9.758	9.504	0.441	0.328	0.254	0.00016	0.00269
IL-22BP	Interleukin-22 receptor subunit alpha-2	Q969J5	11.042	10.788	0.664	0.538	0.254	0.00148	0.00961
WFKN1	WAP, kazal, immunoglobulin, kunitz and NTR domain-containing protein 1	Q96NZ8	9.305	9.050	0.424	0.402	0.255	0.00030	0.00363
C8	Complement component C8	P07357,P07358,P07360	10.578	10.322	0.447	0.421	0.256	0.00032	0.00379
ATS13	A disintegrin and metalloproteinase with thrombospondin motifs 13	Q76LX8	11.806	11.546	0.469	0.500	0.259	0.00089	0.00675
Cathepsin V	Cathepsin L2	O60911	10.427	10.166	0.530	0.596	0.261	0.00048	0.00459
SOST	Sclerostin	Q9BQB4	12.975	12.709	0.611	0.578	0.266	0.00283	0.01455
GRB2-related adapter protein 2	GRB2-related adapter protein 2	O75791	9.739	9.472	0.771	0.431	0.267	0.00056	0.00505
RNF43	E3 ubiquitin-protein ligase RNF43	Q68DV7	10.273	10.003	0.711	0.314	0.270	0.00006	0.00168
Kallikrein 7	Kallikrein-7	P49862	10.465	10.193	0.562	0.583	0.272	0.00419	0.01945
Ephrin-A3	Ephrin-A3	P52797	10.855	10.582	0.558	0.387	0.273	0.00024	0.00325
Lymphotoxin	Tumor necrosis	P36941	11.315	11.040	0.607	0.441	0.275	0.00206	0.01171

b R	factor receptor superfamily member 3								
Angiostatin	Angiostatin	P00747	14.576	14.294	0.394	0.402	0.282	0.00002	0.00072
Tropomyosin 2	Tropomyosin beta chain	P07951	9.453	9.162	0.645	0.507	0.292	0.00393	0.01847
F107B	Protein FAM107B	Q9H098	9.922	9.629	0.440	0.326	0.293	0.00001	0.00055
IL-17 RC	Interleukin-17 receptor C	Q8NAC3	11.038	10.730	0.852	1.013	0.308	0.00188	0.01110
FN1.4	Fibronectin Fragment 4	P02751	16.854	16.542	0.450	0.421	0.312	0.00001	0.00067
FN1.3	Fibronectin Fragment 3	P02751	12.353	12.038	0.697	0.573	0.315	0.00320	0.01595
Myoglobin	Myoglobin	P02144	11.372	11.045	0.526	0.580	0.327	0.00014	0.00248
ApoM	Apolipoprotein M	O95445	13.046	12.706	0.534	0.696	0.340	0.00054	0.00490
Cathepsin S	Cathepsin S	P25774	9.906	9.566	0.376	0.340	0.341	0.00000	0.00000
Carbonic Anhydrase IV	Carbonic anhydrase 4	P22748	9.707	9.365	0.534	0.408	0.342	0.00003	0.00101
RAD51	DNA repair protein RAD51 homolog 1	Q06609	10.584	10.231	0.513	0.573	0.353	0.00001	0.00055
OPG	Tumor necrosis factor receptor superfamily member 11B	O00300	12.251	11.894	0.788	0.573	0.358	0.00238	0.01289
Fibronectin	Fibronectin	P02751	14.963	14.587	0.813	0.700	0.376	0.00158	0.01004
NXPH1	Neurexophilin-1	P58417	14.440	14.054	0.609	0.382	0.385	0.00001	0.00055
ASAH2	Neutral ceramidase	Q9NR71	11.768	11.356	0.896	0.894	0.413	0.00049	0.00463
Thrombin	Thrombin	P00734	11.818	11.392	1.231	1.422	0.425	0.00995	0.03662
Ferritin	Ferritin	P02794 P02792	14.457	13.990	1.167	1.159	0.467	0.01115	0.03947
Leptin	Leptin	P41159	12.338	11.850	1.194	1.019	0.488	0.00879	0.03375

Chk2	Serine/threonine-protein kinase Chk2	O96017	8.052	7.540	1.390	0.777	0.513	0.00990	0.03662
C6	Complement component C6	P13671	14.340	13.805	1.169	0.806	0.534	0.00441	0.02023
SORC2	VPS10 domain-containing receptor SorCS2	Q96PQ0	9.610	9.070	1.347	0.938	0.539	0.00156	0.00997
PTK6	Protein-tyrosine kinase 6	Q13882	10.046	9.476	0.652	0.818	0.570	0.00000	0.00051
C3b	Complement C3b	P01024	17.183	16.518	0.591	0.701	0.665	0.00000	0.00000
LEAP-1	Hepcidin	P81172	13.472	12.755	1.177	1.709	0.717	0.00896	0.03421

*Protein symbol, full protein name and UniProt ID are provided from the UniProtKB database (https://www.uniprot.org). Data were log2 transformed. Mann-Whitney-Wilcoxon tests were performed and adjusted for multiple testing using the Benjamini-Hochberg False Discovery Rate (FDR)-controlling procedure.

Supplementary Table S2 online. Public knowledge resources accessed through EdgeBox and integrated in the INCA molecular interaction network model.

**Resource**	**Version**	**URL**	**Type**<br>**N=nodes, E=edges**
**ChEBI**	v140	https://www.ebi.ac.uk/chebi	N
**ChEMBL**	v21	https://www.ebi.ac.uk/chembl	E
**ENCODE**	2012-09-06	http://encodenets.gersteinlab.org	E
**Ensembl Genes**	Release 84	http://www.ensembl.org	N
**Microcosm**	v5	http://ebi.ac.uk/enright-srv/microcosm	E
**miRBase**	v21	http://mirbase.org	N
**miRecords**	v4	http://c1.accurascience.com/miRecords	E
**miRTarBase**	v6.1	http://mirtarbase.mbc.nctu.edu.tw	E
**Reactome**	v56	http://www.reactome.org	E
**STRING**	v10.0	http://string-db.org	E
**STITCH**	v4.0	http://stitch.embl.de	E
**TFe**	2016-06-09	http://www.cisreg.ca/cgi-bin/tfe/home.pI	E
**WikiPathways**	2016-06-09	http://www.wikipathways.org	E

ChEBI : Chemical Entities of Biological Interest, ChEMBL : Chemical European Molecular Biology Laboratory, ENCODE : Encyclopedia of DNA Elements, STRING : *Search Tool for the Retrieval of Interacting Genes/Proteins*, TFe : transcription factor encyclopedia

Supplementary Table S3 online. Number of edges per edge type in the INCA network model.

**Edge type**	**Number of edges with type**
in_neighbouring_reaction	4956
interacts_with	4507
mirna_targets	4306
in_same_reaction	3388
in_same_component	1749
catalyzes	1051
regulates_transcription	1043
binding	1003
catalysis	363
reaction	250
in_group	232
inhibition	88
inhibits	87
activation	51
correlates_positive	46
expression	13
mediates_interaction	13
modifies_protein	10
activates_transcription	2
drug_targets	1

Supplementary Table S4 online. List of the identified clusters in the INCA network model.

**Cluster number**	**Nodes**	**Edges**	**INCA variables ***	**Top pathways ≠**
1	203	787	BTK, CAPN1, CAPNS1, CAST, *CRK*, CSK, FN1, GRAP2, LYN, PAK6, PRKCA, PRKCB, PRKC1, PRKCZ, *RAC1*, *SRC*, YES1	Immune response activating cell surface receptor signaling pathway; Fc receptor mediated stimulatory signaling pathway; Fc gamma receptor signaling pathway involved in phagocytosis
2	190	625	BAD, GSK3A, GSK3B, *HSP90AA1*, PRKACA, SFN, YWHAE, YWHAG, YWHAH, YWHAQ, *YWHAZ*	Anchoring of the basal body to the plasma membrane; AURKA Activation by TPX2; Regulation of PLK1 Activity at G2/M Transition
3	140	1020	ADRBK1, APP, **C3**, CCL5, CXCL1, CXCL11, CXCL2, CXCL3, CXCL6, CXCL8, F2, PF4, PPBP	Class A/1 (Rhodopsin-like receptors); G alpha (i) signalling events; GPCR ligand binding
4	128	981	CDC37, EREG, FGF16, FGF6, FGF7, FGF8, IL2RG, IL3RA, PDGFA, PDGFB, THPO, YWHAB	activation of MAPKK activity; insulin receptor signaling pathway; fibroblast growth factor receptor signaling pathway
5	141	344	CHEK2, *MAPK14*, MAPK8, MAPKAPK2, RAN, UBE2N	PRC2 methylates histones and DNA; DNA methylation; Meiotic recombination
6	109	209	AKR7A2, HMGB1, HSPD1, **MAPKAPK5**, SORCS2	MyD88 deficiency (TLR2/4); IRAK4 deficiency (TLR2/4); Diseases of Immune System

7	116	334	EEF1B2, EIF4H, N6AMT1, RPS3A, RPS7	Translation; Eukaryotic Translation Initiation; Cap-dependent Translation Initiation
8	71	177	APOM, CLIC1, CTSA, GP6, HAMP, KLK7, KPNB1, MB, RBM39, SBDS, SERPINE2, SGTA, UBE2L3	positive regulation of DNA templated transcription, initiation; regulation of DNA-templated transcription, initiation
9	92	214	**CTSS**, PSMA6, PSMD7, *RPS27A*	APC/C-mediated degradation of cell cycle proteins; Regulation of mitotic cell cycle; anaphase-promoting complex-dependent proteasomal ubiquitin-dependent Protein catabolic process
10	102	199	GDI2, RAC3, *VAV1*	Rho GTPase cycle; Signaling by Rho GTPases; regulation of small GTPase mediated signal transduction
11	82	115	IL17RC, *STAT1*, STAT3	regulation of tyrosine phosphorylation of Stat1 protein; positive regulation of JAK-STAT cascade; JAKSTAT cascade
12	64	107	SMAD2, SMAD3	transmembrane receptor protein serine/threonine kinase signaling pathway; response to transforming growth factor beta; Signaling by TGF-beta Receptor Complex

13	51	95	CMA1, DHH, JAM3, **MMP1**, **MMP**7, PLG, SELP, TNFRSF11B	Extracellular matrix organization; extracellular matrix organization; extracellular structure organization
14	61	96	RPS6KA3, RPS6KA5	stress-activated protein kinase signaling cascade; stress-activated MAPK cascade; Recycling pathway of L1
15	52	111	CAMKK1, PRKAA2, PRKAB2, PRKAG1	PKB-mediated events; mTOR signalling; Energy dependent regulation of mTOR by LKB1-AMPK
16	67	101	ISG15, PPIE, SSRP1	Dual incision in TC-NER; Gap-filling DNA repair synthesis and ligation in TCNER; Transcription-Coupled Nucleotide Excision Repair (TC-NER)
17	61	74	ING1, RAD51	double-strand break repair via homologous recombination; recombinational repair; Homology Directed Repair
18	49	86	CSNK2A1, CSNK2A2, CSNK2B, SPHK1	Chaperonin-mediated protein folding; Protein folding; Association of TriC/CCT with target proteins during biosynthesis
19	53	70	NME1-NME2, PTK6, THBS1	

20	53	93	ADIPOQ, LEP	Transcriptional regulation of white adipocyte differentiation; PPARA activates gene expression; Regulation of lipid metabolism by Peroxisome proliferator-activated receptor alpha (PPARalpha)
21	49	72	EFNA3, HNRNPAB, PKM	
22	46	56	CASP3, EIF4G2	Apoptosis; Programmed Cell Death; Apoptotic execution phase
23	40	52	BCL2, BCL2L1, NMT1	Intrinsic Pathway for Apoptosis; Apoptosis; Programmed Cell Death
24	49	57	DDX19B, IDE	
25	41	75	AREG, FTH1, FTL, NAPA	Golgi vesicle transport; trans-Golgi Network Vesicle Budding; Clathrin derived vesicle budding
26	41	49	TPM2, TPM4	Striated Muscle Contraction; muscle filament sliding; actinmyosin filament sliding
27	33	75	ADAMTS13, C6, C8A, C8B, C8G	Complement cascade; complement activation; Regulation of Complement cascade
28	26	45	HSD17B10, IMPDH1, LDHB, MDH1, PPA1	

29	39	45	NUDCD3, UFM1	
30	28	33	HSP90AB1, STUB1	response to unfolded protein; Cellular response to heat stress; Regulation of HSF1-mediated heat shock response
31	23	26	PDE11A, PDE5A	Nitric oxide stimulates guanylate cyclase; cGMP metabolic process; Platelet homeostasis
33	26	28	EDAR, TPI1	
37	21	23	PA2G4, PDIA3	Antigen Presentation: Folding, assembly and peptide loading of class I MHC; ER-Phagosome pathway; antigen processing and presentation of exogenous peptide antigen via MHC class I, TAP-dependent
40	16	29	DKK1, KREMEN2, SOST	regulation of canonical Wnt signaling pathway; canonical Wnt signaling pathway; regulation of Wnt signaling pathway
42	11	15	OLFM4, RNF43, RSPO4, SMPDL3A, WFIKKN1	Regulation of FZD by ubiquitination; Budding and maturation of HIV virion; Membrane binding and targetting of GAG proteins
44	17	29	CA13, CA4	Pyruvate metabolism and Citric Acid (TCA) cycle; The citric

				acid (TCA) cycle and respiratory electron transport; tricarboxylic acid cycle
48	17	20	METAP1, METAP2	
50	13	15	PRDX1, PRDX6	Detoxification of ReactiveOxygen Species
51	14	18	NPPA, SNCA	Amyloid fiber formation
56	13	17	ENO1, ENO2	Gluconeogenesis; Glucose metabolism; Gluconeogenesis
unclustered	431	503	ACP1, ADGRE5, AK1, ARID3A, ASAH2, CPNE1, CTSV, CYP3A4, DKK4, EIF5A, EPB41, ESD, **FAM107B**, FER, FLT3, HIST1H1C, IL22RA2, LTBR, MDK, NACA, NSFL1C, NXPH1, PDXP, PEBP1, PPID, PPIF, SEZ6L2, SFRP1, SFTPD, SNX4, STIP1, TKT, TPT1, VTA1, XPNPEP1	

*INCA variables in bold indicate the 6 proteins identified to discriminate between HF patients who died or alive after 3 years follow-up; INCA variables in italic indicate the top 10 nodes with the highest betweenness centrality calculated from the INCA network; [≠]Information provided by GO (Gene Ontology, http://geneontology.org/).

Supplementary Table S5 online. List of the proteins in the INCA network linked with the 6 proteins selected by LASSO analysis.

Protein*	INCA nodes /total linked nodes	Linked INCA nodes (Uniprot ID)/cluster**	Fold-change (Case vs. Control, mean ± SEM)***	*P* value≠	GO associated terms≡
**CTAS**	1/91	CATL2<br>Cathepsin L2<br>(O60911)/-	0.79<br>(1155 ± 377.1 *vs* 1467 ± 510.5)	$5.31\ 10^{-5}$	-
**MAPK5**	2/114	KAPCA<br>cAMP-dependent protein kinase catalytic subunit alpha<br>(P17612)/2	1.79<br>(10809 ± 8410 *vs* 6032 ± 4931)	$2.07\ 10^{-4}$	Anchoring of the basal body to the plasma membrane; AURKA Activation by TPX2; Regulation of PLK1 Activity at G2/M Transition; Loss of Nlp from mitotic centrosomes; Recruitment of mitotic centrosome proteins and complexes
		MK14<br>Mitogen-activated protein kinase 14<br>(Q16539)/5	1.31<br>(1472 ± 763.1 *vs* 1127 ± 576.1)	0.0024	PRC2 methylates histones and DNA; DNA methylation; Meiotic recombination; DNA methylation on cytosine; SIRT1 negatively regulates rRNA Expression
**C3**	12/229	PLF4<br>Platelet factor 4<br>(P02776)/3	2.14<br>(85972 ± 63555 *vs* 40163 ± 34472)	$4.32\ 10^{-6}$	
		CXCL3<br>C-X-C motif chemokine 3<br>(P19876)/3	1.40<br>(404.2 ± 179.6 *vs* 288,1 ± 134.8)	$8.25\ 10^{-6}$	
		CXCL7<br>Platelet basic protein<br>(P02775)/3	1.60<br>(11124 ± 7332 *vs* 6967 ± 5033)	$1.71\ 10^{-4}$	
		CXCL1<br>Growth-regulated alpha	1.22<br>(2466 ± 761.9 *vs* 2017 ± 653.9)	$6.52\ 10^{-5}$	

		protein (P09341)/3			
		CCL5 C-C motif chemokine 5 (P13501)/3	1.56 (38821 ± 24401 *vs* 24812 ± 18433)	$9.03\ 10^{-5}$	Class A/1 (Rhodopsin-like receptors); G alpha (i) signalling events; GPCR ligand binding; Peptide ligand-binding receptors; G alpha (q) signalling events
		CXCL6 C-X-C motif chemokine 6 (P80162)/3	1.34 (1119 ± 463.4 *vs* 832.8 ± 428)	$1.95\ 10^{-5}$	
		IL8 Interleukin-8 (P10145)/3	1.14 (676.5 ± 186.3 *vs* 591.6 ± 193.6)	0.0012	
		CXCL11 C-X-C motif chemokine 11 (O14625)/3	1.18 (4157 ± 1683 *vs* 3537 ± 1365)	0.0178	
		SDF1 Stromal cell-derived factor 1 (P48061)/3	1.10 (3872 ± 822.2 *vs* 3513 ± 644.8)	0.0016	
		APP Amyloid-beta precursor protein (P05067)/3	1.35 (47479 ± 24460 *vs* 35111 ± 19466)	0.0011	
		STAT1 Signal transducer and activator of transcription 1-alpha/beta (P42224)/11	1.57 (6437 ± 3912 *vs* 4100 ± 2840)	$4.64\ 10^{-5}$	Regulation of tyrosine phosphorylation of Stat1 protein; positive regulation of JAK-STAT cascade; JAK-STAT cascade; tyrosine phosphorylation of Stat1 protein; positive regulation of cytokine production
		C6 Complement component C6	0.40 (11344 ± 3221 *vs* 28437 ± 21843)	$5.61\ 10^{-6}$	Complement cascade; complement activation; Regulation of Complement cascade; regulation of complement activation; complement

		(P13671)/27			activation, classical pathway
**MMP1**	4/39	PLMN<br>Plasminogen<br>(P00747)/13	0.83<br>(20606 ± 5462 *vs* 24685 ± 5986)	1.41 10^{-5}	Extracellular matrix organization; extracellular matrix organization; extracellular structure organization; Activation of Matrix Metalloproteinases; Degradation of the extracellular matrix
		CMA1<br>Chymase<br>(P23946)/13	1.28<br>(627.5 ± 182.4 *vs* 490.2 ± 94.76)	2.40 10^{-8}	
		MMP7<br>Matrilysin<br>(P09237)/13	1.44<br>(913.1 ± 51.6 *vs*. 635.1 ± 41.5)	0.0011	
		BCL2<br>Apoptosis regulator Bcl-2<br>(P10415)/23	1.36<br>(1289 ± 432.8 *vs* 946.4 ± 283.7	8.69 10^{-8}	Intrinsic Pathway for Apoptosis; Apoptosis; Programmed Cell Death; mitochondrial membrane organization; regulation of mitochondrial membrane permeability
**MMP7**	2/36	MMP1<br>Interstitial collagenase<br>(P03956)/13	1.96<br>(1499.6 ± 93.7 *vs*. 766.4 ± 40.2)	5.49 10^{-5}	Extracellular matrix organization; extracellular matrix organization; extracellular structure organization; Activation of Matrix Metalloproteinases; Degradation of the extracellular matrix
		CATL2<br>Cathepsin L2<br>(O60911)/-	0.79<br>(1155 ± 377.1 *vs* 1467 ± 510.5)	5.31 10^{-5}	**-**
**F107B**	0/15	-	-	-	-

* Selected protein candidates from Table 4; **Full name, protein symbol and UniProt ID are provided from UniProtKB database (https://www.uniprot.org/); -, no cluster and information available in the INCA network; *** Data are expressed in relative fluorescence unit; ≠*P* value was calculated by Mann-Whitney-Wilcoxon test; ≡ Information provided by GO (Gene Ontology, http://geneontology.org/).

Supplementary Table S6 online. Normalization scale factors

PlateId	SampleId	SampleGroup	HybControlNormScale	NormScale.40	NormScale.1	NormScale.0.005	RowCheck
Set 01	341	Control	0.954	1.078	1.129	0.997	PASS
Set 01	123	Dead	0.961	0.922	1.028	0.822	PASS
Set 01	1071	Control	0.895	1.381	1.305	1.105	PASS
Set 01	30	Dead	0.924	0.864	0.789	1.082	PASS
Set 01	284	Control	0.993	0.957	0.965	0.968	PASS
Set 01	96	Dead	0.988	0.887	0.894	1	PASS
Set 01	231	Control	0.953	0.549	0.734	1.006	PASS
Set 01	828	Control	0.951	0.875	0.778	0.818	PASS
Set 01	265	Control	0.904	1.156	1.049	0.978	PASS
Set 01	546	Control	0.98	1.12	1.057	0.915	PASS
Set 01	769	Control	0.909	0.598	0.637	0.848	PASS
Set 01	50	Dead	1.001	0.79	0.779	0.788	PASS
Set 01	294	Dead	0.953	0.898	1.321	1.125	PASS
Set 01	185	Dead	0.996	1.001	0.866	1.006	PASS
Set 01	329	Control	0.95	0.86	1.004	0.936	PASS
Set 01	318	Dead	0.939	1.046	1.02	1.022	PASS
Set 01	545	Control	0.945	1.107	1.207	1.231	PASS
Set 01	963	Control	1.043	1.034	0.974	0.826	PASS
Set 01	704	Control	1.062	1.122	1.171	0.876	PASS
Set 01	130	Dead	0.992	1.206	1.103	0.931	PASS
Set 01	82	Dead	0.931	1.154	1.084	1.253	PASS
Set 01	217	Dead	0.953	0.832	0.905	0.922	PASS
Set 01	782	Control	0.912	1.342	1.388	1.063	PASS
Set 01	247	Dead	0.882	0.851	0.926	1.019	PASS
Set 01	94	Dead	0.905	1.095	1.071	1.051	PASS
Set 01	1008	Control	0.93	1.332	1.225	1.099	PASS
Set 01	772	Dead	0.971	1.294	1.298	1.167	PASS
Set 01	1018	Dead	0.982	1.106	1.009	1.065	PASS
Set 01	59	Dead	0.949	0.793	0.771	0.936	PASS
Set 01	102	Dead	0.976	0.857	0.833	0.993	PASS
Set 01	969	Control	0.916	0.701	0.714	0.705	PASS
Set 01	540	Control	0.961	1.394	1.271	1.097	PASS
Set 01	25	Dead	1.032	0.867	0.94	1.011	PASS
Set 01	384	Control	0.977	1.226	1.13	1.088	PASS

Set 01	812	Control	0.923	0.894	0.818	0.817 PASS
Set 01	93	Dead	0.942	0.913	0.919	0.912 PASS
Set 01	656	Control	0.912	0.955	0.938	0.969 PASS
Set 01	331	Dead	0.893	0.637	0.901	0.933 PASS
Set 01	14	Dead	0.996	1.129	0.972	0.976 PASS
Set 01	694	Control	0.968	0.212	0.578	0.762 FAIL
Set 01	201	Dead	0.902	0.727	0.839	0.943 PASS
Set 01	1012	Control	0.92	1.102	1.12	1.151 PASS
Set 01	58	Dead	1.031	0.783	0.937	0.998 PASS
Set 01	24	Dead	0.99	1.336	1.167	1.142 PASS
Set 01	234	Dead	0.923	0.446	0.694	0.946 PASS
Set 01	120	Control	0.961	1.164	1.082	1.011 PASS
Set 01	333	Dead	1.09	0.794	0.881	0.981 PASS
Set 01	375	Control	0.995	1.133	0.975	0.9 PASS
Set 01	378	Dead	0.943	0.99	0.996	1.089 PASS
Set 01	81	Dead	1.013	1.171	1.05	1.075 PASS
Set 01	288	Control	0.933	1.255	1.161	1.037 PASS
Set 01	29	Dead	0.915	1.098	1.018	1.05 PASS
Set 01	1062	Control	0.931	1.081	1.05	0.969 PASS
Set 01	38	Dead	1.062	0.473	0.627	0.805 PASS
Set 01	54	Dead	1.01	0.467	0.68	0.818 PASS
Set 01	357	Control	0.951	0.925	1.082	0.943 PASS
Set 01	516	Control	1.073	1.444	1.521	1.177 PASS
Set 01	503	Control	0.993	0.917	0.977	0.89 PASS
Set 01	88	Dead	1.002	0.608	0.772	0.914 PASS
Set 01	538	Control	1.115	1.012	1.081	1.003 PASS
Set 01	945	Control	1.056	1.047	0.953	0.893 PASS
Set 01	818	Control	1.043	1.134	1.138	1.153 PASS
Set 01	839	Control	1.055	1.322	1.095	1.098 PASS
Set 01	970	Control	1.012	1.012	1.143	0.922 PASS
Set 01	1219	Control	0.977	1.348	1.254	1.23 PASS
Set 01	118	Dead	0.974	1.009	0.999	0.944 PASS
Set 01	139	Control	0.965	0.944	0.897	0.925 PASS
Set 01	1212	Control	1.071	1.739	1.886	1.703 PASS
Set 01	116	Dead	1.098	1.186	1.071	1.124 PASS
Set 01	27	Control	1.024	0.828	0.827	0.983 PASS

Set 01	1047	Dead	1.009	1.077	1.061	0.995	PASS
Set 01	636	Control	0.968	1.118	1.036	1.002	PASS
Set 01	267	Control	0.951	1.298	1.307	1.152	PASS
Set 01	362	Dead	0.969	1.237	1.232	1.171	PASS
Set 01	396	Dead	1.013	1.013	1.016	0.996	PASS
Set 01	31	Dead	0.98	0.753	0.74	1.001	PASS
Set 01	554	Control	0.985	1.001	0.982	1.153	PASS
Set 01	297	Dead	1.111	1.166	1.145	1.016	PASS
Set 01	585	Control	1.049	1.183	1.165	1.003	PASS
Set 01	383	Control	1.008	1.285	1.361	1.143	PASS
Set 01	48	Dead	1.043	0.863	0.868	0.927	PASS
Set 01	968	Control	1.07	1.242	1.371	1.238	PASS
Set 01	84	Dead	1.07	0.61	0.56	0.89	PASS
Set 01	76	Control	1.119	0.864	0.851	1.016	PASS
Set 02	1007	Control	0.978	1.028	1.084	1.225	PASS
Set 02	831	Control	1.015	0.381	0.573	0.745	FAIL
Set 02	861	Control	1.031	1.35	1.498	1.054	PASS
Set 02	728	Dead	0.961	1.063	1.197	0.974	PASS
Set 02	566	Control	1.066	1.357	1.222	1.046	PASS
Set 02	340	Control	1.046	0.63	0.727	0.893	PASS
Set 02	815	Control	1.086	0.796	0.863	0.81	PASS
Set 02	422	Dead	0.971	1.05	1.003	0.992	PASS
Set 02	372	Dead	1.009	1.255	1.121	1.289	PASS
Set 02	264	Dead	1.02	0.617	0.791	1.003	PASS
Set 02	583	Control	1.063	1.185	0.946	0.812	PASS
Set 02	46	Dead	0.964	1.192	1.036	1	PASS
Set 02	44	Dead	1.02	1.013	0.988	0.988	PASS
Set 02	486	Control	1.02	1.306	1.236	1.1	PASS
Set 02	360	Dead	0.996	1.017	0.9	0.924	PASS
Set 02	136	Dead	0.976	1.19	1.126	0.962	PASS
Set 02	432	Control	1.012	0.84	0.861	1.071	PASS
Set 02	150	Dead	0.963	1.024	1.056	1.165	PASS
Set 02	149	Dead	0.982	1.035	0.99	0.913	PASS
Set 02	189	Dead	0.939	1.017	1.054	1.057	PASS
Set 02	778	Control	1.019	183.002	1.116	1.412	FAIL
Set 02	291	Dead	1.023	1	1.145	1.064	PASS

Set 02	997	Control	0.992	1.283	1.061	0.963	PASS
Set 02	796	Control	1.012	1.337	1.184	1.093	PASS
Set 02	387	Dead	1.035	0.825	0.796	0.836	PASS
Set 02	211	Dead	1.043	0.798	0.899	0.969	PASS
Set 02	374	Control	1.052	1.205	1.09	1.004	PASS
Set 02	66	Dead	1.019	0.578	0.744	0.871	PASS
Set 02	834	Control	0.948	1.119	1.207	1.218	PASS
Set 02	37	Dead	0.943	1.116	1.015	0.974	PASS
Set 02	72	Dead	0.932	0.819	0.846	1.011	PASS
Set 02	346	Control	1.039	1.629	1.582	1.531	PASS
Set 02	154	Dead	1.059	0.983	1.063	0.953	PASS
Set 02	111	Dead	0.977	1.093	0.998	0.893	PASS
Set 02	334	Dead	0.977	1.221	1.35	1.157	PASS
Set 02	274	Control	1.071	0.835	0.986	0.99	PASS
Set 02	737	Control	1.117	1.07	1.064	1.003	PASS
Set 02	108	Dead	1.049	0.85	1.01	1.083	PASS
Set 02	8	Dead	1.091	1.005	0.972	1.045	PASS
Set 02	648	Control	1.1	0.098	0.298	0.619	FAIL
Set 02	678	Control	1.149	1.174	1.116	0.967	PASS
Set 02	550	Control	1.062	1.306	1.178	1.09	PASS
Set 02	489	Control	0.969	1.181	1.122	0.973	PASS
Set 02	105	Dead	1.05	0.812	0.897	0.908	PASS
Set 02	999	Dead	1.107	0.91	0.967	0.976	PASS
Set 02	17	Dead	1.073	0.845	0.94	0.953	PASS
Set 02	412	Dead	1.047	0.937	0.976	1.045	PASS
Set 02	368	Dead	1.029	1.345	1.335	1.066	PASS
Set 02	2	Dead	1.075	0.701	0.796	0.801	PASS
Set 02	702	Control	0.971	1.256	1.197	1.101	PASS
Set 02	12	Dead	1.001	0.794	0.768	0.922	PASS
Set 02	33	Dead	0.987	0.631	0.78	0.964	PASS
Set 02	292	Dead	0.966	1.324	1.298	1.21	PASS
Set 02	45	Dead	1	1.082	1.052	0.929	PASS
Set 02	917	Control	0.963	0.948	0.967	1.003	PASS
Set 02	152	Dead	0.983	1.184	1.135	1.215	PASS
Set 02	623	Control	0.953	1.298	1.016	1.04	PASS
Set 02	161	Dead	0.96	0.512	0.638	0.886	PASS

Set 02	214	Dead	0.988	0.722	0.799	0.898	PASS
Set 02	883	Control	0.945	1.221	1.18	1.124	PASS
Set 02	390	Control	1.058	1.028	0.988	1.003	PASS
Set 02	43	Dead	0.981	0.51	0.627	0.891	PASS
Set 02	498	Control	1.009	1.41	1.356	1.205	PASS
Set 02	864	Control	1.026	0.976	1.005	1.007	PASS
Set 02	851	Control	1.017	1.07	1.095	1.093	PASS
Set 02	892	Control	1.035	0.987	1.126	0.975	PASS
Set 02	86	Control	1.101	0.589	0.783	0.772	PASS
Set 02	947	Control	1.048	0.861	0.934	0.767	PASS
Set 02	354	Control	1.01	1.51	1.307	1.063	PASS
Set 02	282	Dead	1.064	0.933	0.859	0.92	PASS
Set 02	75	Dead	1.02	1.011	0.981	1.058	PASS
Set 02	293	Dead	1.019	0.781	0.748	0.941	PASS
Set 02	273	Control	1.005	0.729	0.756	0.932	PASS
Set 02	660	Control	1.066	0.893	0.864	0.887	PASS
Set 02	940	Control	0.983	1.674	1.825	1.817	PASS
Set 02	92	Dead	0.98	0.875	0.993	1.03	PASS
Set 02	460	Control	0.971	1.181	1.17	1.052	PASS
Set 02	339	Control	1.08	0.998	0.929	1.123	PASS
Set 02	133	Dead	1.101	0.815	0.83	0.85	PASS
Set 02	552	Control	1.107	1.123	1.058	1.136	PASS
Set 02	53	Dead	1.052	0.683	0.77	0.958	PASS
Set 02	151	Dead	1.196	0.981	1.01	0.937	PASS
Set 02	590	Dead	1.143	1.356	1.184	1.115	PASS
Set 02	781	Control	1.093	1.194	1.293	1.014	PASS

Samples failing the intra-run normalization scael factors are highlited in yellow
